# C-di-GMP Regulates Motile to Sessile Transition by Modulating MshA Pili Biogenesis and Near-Surface Motility Behavior in *Vibrio cholerae*

**DOI:** 10.1371/journal.ppat.1005068

**Published:** 2015-10-27

**Authors:** Christopher J. Jones, Andrew Utada, Kimberly R. Davis, Wiriya Thongsomboon, David Zamorano Sanchez, Vinita Banakar, Lynette Cegelski, Gerard C. L. Wong, Fitnat H. Yildiz

**Affiliations:** 1 Department of Microbiology and Environmental Toxicology, University of California, Santa Cruz, Santa Cruz, California, United States of America; 2 Department of Bioengineering, Department of Chemistry and Biochemistry, California Nano Systems Institute, University of California, Los Angeles, Los Angeles, California, United States of America; 3 Department of Chemistry, Stanford University, Stanford, California, United States of America; University of Washington, UNITED STATES

## Abstract

In many bacteria, including *Vibrio cholerae*, cyclic dimeric guanosine monophosphate (c-di-GMP) controls the motile to biofilm life style switch. Yet, little is known about how this occurs. In this study, we report that changes in c-di-GMP concentration impact the biosynthesis of the MshA pili, resulting in altered motility and biofilm phenotypes in *V*. *cholerae*. Previously, we reported that *cdgJ* encodes a c-di-GMP phosphodiesterase and a Δ*cdgJ* mutant has reduced motility and enhanced biofilm formation. Here we show that loss of the genes required for the mannose*-*sensitive hemagglutinin *(*MshA*)* pilus biogenesis restores motility in the Δ*cdgJ* mutant. Mutations of the predicted ATPase proteins *mshE* or *pilT*, responsible for polymerizing and depolymerizing MshA pili, impair near surface motility behavior and initial surface attachment dynamics. A Δ*cdgJ* mutant has enhanced surface attachment, while the Δ*cdgJmshA* mutant phenocopies the high motility and low attachment phenotypes observed in a Δ*mshA* strain. Elevated concentrations of c-di-GMP enhance surface MshA pilus production. MshE, but not PilT binds c-di-GMP directly, establishing a mechanism for c-di-GMP signaling input in MshA pilus production. Collectively, our results suggest that the dynamic nature of the MshA pilus established by the assembly and disassembly of pilin subunits is essential for transition from the motile to sessile lifestyle and that c-di-GMP affects MshA pilus assembly and function through direct interactions with the MshE ATPase.

## Author Summary

The human pathogen *Vibrio cholerae* causes the debilitating disease cholera through ingestion of contaminated food and water. *V*. *cholerae* is a natural inhabitant of aquatic environments. Transmission of *V*. *cholerae* to the human host is dependent on survival of the pathogen in aquatic reservoirs where it is challenged with many stressors, including changes in the physiochemical parameters of environments and predation by protozoa and phages. One method utilized to endure these assaults is to form a multicellular community called a biofilm. The signaling molecule cyclic dimeric guanosine monophosphate (c-di-GMP) is utilized to induce biofilm formation in many bacteria, including *V*. *cholerae*. We demonstrate that c-di-GMP promotes the production of a cell surface structure called MshA pili by binding the molecular motor responsible for polymerizing pilus subunits. These pili are adhesive appendages that are essential for attachment to surfaces. This study identifies a novel mechanism for c-di-GMP regulation of pilus production through interactions with the molecular motor responsible for pilus assembly. Since many bacteria utilize pili for attachment to surfaces and c-di-GMP as a pro-biofilm signaling molecule, the mechanism for pilus regulation and biofilm formation described here may be widespread among many pathogens.

## Introduction

*Vibrio cholerae*, the causative agent of the human intestinal disease cholera, is a natural inhabitant of aquatic ecosystems [1]. Cholera infection results from consumption of food and water contaminated with *V*. *cholerae*. Subsequently the bacteria turn on regulatory networks that facilitate bacterial growth and survival during the infection process [2]. They also activate production of virulence factors including the toxin-coregulated pilus (TCP), essential for intestinal colonization, and the cholera toxin (CT), responsible for production of massive watery diarrhea that results in dissemination of *V*. *cholerae* back to aquatic ecosystems [3]. *V*. *cholerae*’s ability to cause epidemics is tied to its dissemination and survival in aquatic habitats and its transmission to the human host. One critical factor for dissemination, environmental survival and transmission of the pathogen is its ability to form matrix enclosed surface-associated communities termed biofilms [4–6]. *V*. *cholerae* forms biofilms on the surfaces of phytoplankton and zooplankton [7], and exists in the surface waters of cholera endemic areas in matrix-enclosed aggregates thought to arise from biofilm-like populations of *V*. *cholerae* present in human stools [5]. Removal of particles >20 μm in diameter from water can reduce cholera incidence by 48% [8]. Additionally, growth in a biofilm induces a hyper-infectious phenotype [9]. Collectively, these studies highlight the importance of the biofilm growth mode in both the intestinal and aquatic phases of the *V*. *cholerae* life cycle.

Biofilm formation by *V*. *cholerae* begins with surface attachment, and subsequent development of microcolonies and mature biofilm structures [10–16]. The biofilm matrix is primarily composed of Vibrio exopolysaccharide (VPS) [17], extracellular DNA [18], and biofilm matrix proteins (RbmA, RbmC, and Bap1) [13,14,16,19], which are required for cell-cell and cell-surface interactions and development of mature *V*. *cholerae* biofilms [15].

Two cell-surface structures, a single polar flagellum and type IVa mannose-sensitive hemagglutanin pili (MshA), are critical for initial attachment and biofilm formation [11,20]. Type IVa pili have the ability to rapidly extend and retract, contributing to twitching and swarming motility in many bacteria [21]. Though *V*. *cholerae* produces the type IVa MshA pilus, twitching motility has not been reported. Genes required for biogenesis of MshA pilus are clustered into 16.7 kb region in *V*. *cholerae* chromosome-I and organized into two operons: the first operon harbors 9 genes from *mshI-mshF predicted to* encode proteins required for assembly and secretion and the second harboring 7 genes from *mshB—mshQ* encoding pilus structural components *mshB-Q* [22]. The pilus is comprised of repeats of the major pilin, MshA. Predicted function of the proteins encoded by MSHA gene locus are included in Table 1.

**Table 1:** Extragenic suppressors of Δ*cdgJ* motility phenotype.

Gene	Gene #	# of insertions	Homologous Characterized Protein	Cluster Orthologous Group	Percent Identity/ Similarity	Predicted Function
***mshI***	VC0399	6	Gspl from *Vibrio vulnificus*	PilN Superfamily	25/49	
***mshJ***	VC0400	1	PilO from *Pseudomonas aeruginosa*		22/46	
***mshL***	VC0402	3	PilQ Dodecameric Complexes from *Neisseria meningitidis*	Type II Secretin Superfamily	25/46	Outer membrane pore
***mshM***	VC0403	2	ExeA from *Aeromonas hydrophila*	ABC ATPase	43/57	
***mshN***	VC0404	3	Tetratricopeptide repeat domain containing protein from *Psuedomonas aeruginosa*	TPR domain	37/54	
***mshE***	VC0405	1	GspE Hexamer ATPase from *Vibrio cholerae*	ABC ATPase	45/63	Extension ATPase
***mshG***	VC0406	1	Type 4 fimbrial assembly protein PilC from *Pseudomonas aeruginosa*	Type II secretory pathway, component PulF	32/58	Inner membrane pilus platform
***mshA***	VC0409	2	Pilin from *Pseudomonas putida*	major pilin subunit	49/65	Major pilin
***mshC***	VC0410	1	Pilin from *Pseudomonas putida*	Type II secretory pathway, pseudopilin PulG	26/45	Minor pilin
***mshD***	VC0411	2	Pullulanase secretion protein PulG from *Klebsiella pneumoniae*	Type II secretory pathway, pseudopilin PulG	35/48	Minor pilin
***mshO***	VC0412	1	Pilin from *Neisseria meningitides*	Type II secretory pathway, pseudopilin PulG	34/52	Minor pilin
***mshP***	VC0413	1	Pectic enzymes secretion protein OutG from *Dickeya chrysanthemi*		23/46	
***mshQ***	VC0414	2	none			
***pilT***	VC0462	1	PilT from *Pseudomonas*. *aeruginosa*	ABC ATPase	67/82	Retraction ATPase
***cdgK***	VC1104	2	Diguanylate cyclase YdaM from *Escherichia coli*	Class III nucleotidyl cyclases	38/55	diguanylate cyclase
***cdgH***	VC1067	4	Diguanylate cyclase YeaP from *Escherichia coli*	Periplasmic Binding Protein, Class III necleotidyl cyclases	30/52	diguanylate cyclase

MshA pili and flagellum are also crucial for two distinct near-surface motility trajectories of *V*. *cholerae*: ‘roaming’ and ‘orbiting’, [10]. Low curvature ‘roaming’ trajectories meander over large distances and result from weak MshA-surface interactions. In contrast, orbiting trajectories repeatedly trace out tight circular tracks over the same region, and are the result of strong MshA-surface interactions. Cells that attach to the surface come from the orbiting subpopulation, while the roaming subpopulation pass over the surface without attaching. That orbiting motility is ablated when a mannose derivative is added to the medium to saturate MshA pilus binding further indicates that interactions between MshA pili with the surface are important [10].

The second messenger cyclic dimeric guanosine monophosphate (c-di-GMP) is an important promoter of the switch from motile planktonic growth mode to biofilm growth mode [23–25]. c-di-GMP synthesis is catalyzed by diguanylate cyclases (DGC) harboring a GGDEF domain while degradation is catalyzed by phosphodiesterases (PDE) harboring an EAL or HD-GYP domain. Subsequently, c-di-GMP is sensed by different classes of receptor proteins or RNAs and thereby converted to specific phenotypic outputs affecting motility, biofilm formation, and virulence. Elevated intracellular levels of c-di-GMP inhibit motility both by post-transcriptional and transcriptional mechanisms. In *Salmonella enterica* and *Escherichia coli*, the PilZ class of c-di-GMP receptor protein YcgR affects flagellar motor functions through interaction with FliG and FliM subunits of the flagellar rotor or the stator subunit MotA [26,27]. In *Pseudomonas aeruginosa* and *V*. *cholerae*, c-di-GMP inhibits motility by repressing transcription of flagellar genes through the AAA+ ATPase enhancer binding class of c-di-GMP receptor and transcriptional regulators FleQ [28] and FlrA [29], respectively. In addition to the regulation of flagellum production and activity by c-di-GMP, there are reports of c-di-GMP regulating the assembly and activity of Type IV pili. In *Klebsiella pneumoniae*, c-di-GMP is bound by PilZ class of c-di-GMP receptor protein MrkH, which upregulates the transcription of the fimbrial subunit *mrkA* [30–32]. In *P*. *aeruginosa*, the degenerate GGDEF-EAL domain class of c-di-GMP receptor FimX modulates Type IV pili production in an intracellular c-di-GMP concentration-dependent manner [33,34].

The *V*. *cholerae* genome encodes 31 GGDEF domain, 12 EAL domain and 10 dual GGDEF/EAL domain proteins [35]. Systematic analysis of in-frame deletion mutants of all *V*. *cholerae* genes encoding proteins with GGDEF and/or EAL domains for motility phenotypes revealed that four DGCs (CdgH, CdgK, CdgL, and CdgD) and two PDEs (CdgJ and RocS) affect motility in an LB soft agar motility assay [36]. Though deletion of the PDE *cdgJ* affected motility, no difference in intracellular c-di-GMP concentration was observed between the Δ*cdgJ* mutant and WT [36]. This measurement was conducted from a population, so there may be subcellular localized differences or population differences that affect motility. The molecular mechanism of c-di-GMP mediated motility repression and contribution of these DGCs and PDEs to switch from motile to surface-associated lifestyle remains elusive. In this study, we demonstrate that c-di-GMP inversely regulates motility and biofilm formation through direct regulation of the assembly and activity of the MshA pilus. Swimming motility is impaired in strains lacking the phosphodiesterase *cdgJ*, and disruption of the assembly or disassembly of the MshA pilus restores motility to WT levels by reducing the interactions with surfaces. Quantitative measurements indicate that c-di-GMP leads to increased production of MshA pili, which in turn bind surfaces and reduce motility. We demonstrate that the ATPase responsible for pilus polymerization, MshE, functions as a c-di-GMP receptor thereby providing an input for the c-di-GMP signal into the assembly of the MshA pilus. Collectively, this study elucidates how type IV pili and swimming motility are regulated by c-di-GMP in *V*. *cholerae* by presenting the first characterization of the complex involved in the assembly and disassembly of the MshA pilus and how c-di-GMP regulates the production and function of this complex.

## Results

### MshA pilus impacts CdgJ mediated motility

**Fig 1:**
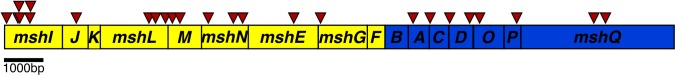
Schematic representation of the transposon insertions in the Msh operons. The operon encoding genes required for assembly and secretion (*mshI-F)* is displayed in yellow and the operon encoding pilus structural components *mshB-Q* is displayed in blue. Transposon insertions are indicated by red arrowheads above the operon. There were no identical insertions observed; arrows are shifted up for clarity indicating two insertions near each other.

CdgJ is a PDE and a *cdgJ* mutant displays a decrease in motility, enhanced VPS production, and increased biofilm formation compared to WT [36]. To begin investigating the mechanism by which CdgJ impacts motility, we performed transposon mutagenesis in a *cdgJ* deletion mutant (Δ*cdgJ*) and screened the resulting mutants for enhanced motility phenotype using LB soft agar motility assay. We screened 7054 transposon mutants and identified 42 extragenic suppressor mutants with increased motility and mapped the transposon insertions site to 22 different genes (Table 1, Fig 1). As previously reported, we found that mutations in the DGC encoding genes *cdgH* and *cdgK* in a Δ*cdgJ* strain enhance motility [36]. We also found that insertion in the gene encoding a pilus retraction motor, PilT, and insertions into different genes predicted to be required in mannose-sensitive hemagglutinin type IV pilus (MshA) biogenesis restored swimming motility.

**Fig 2:**
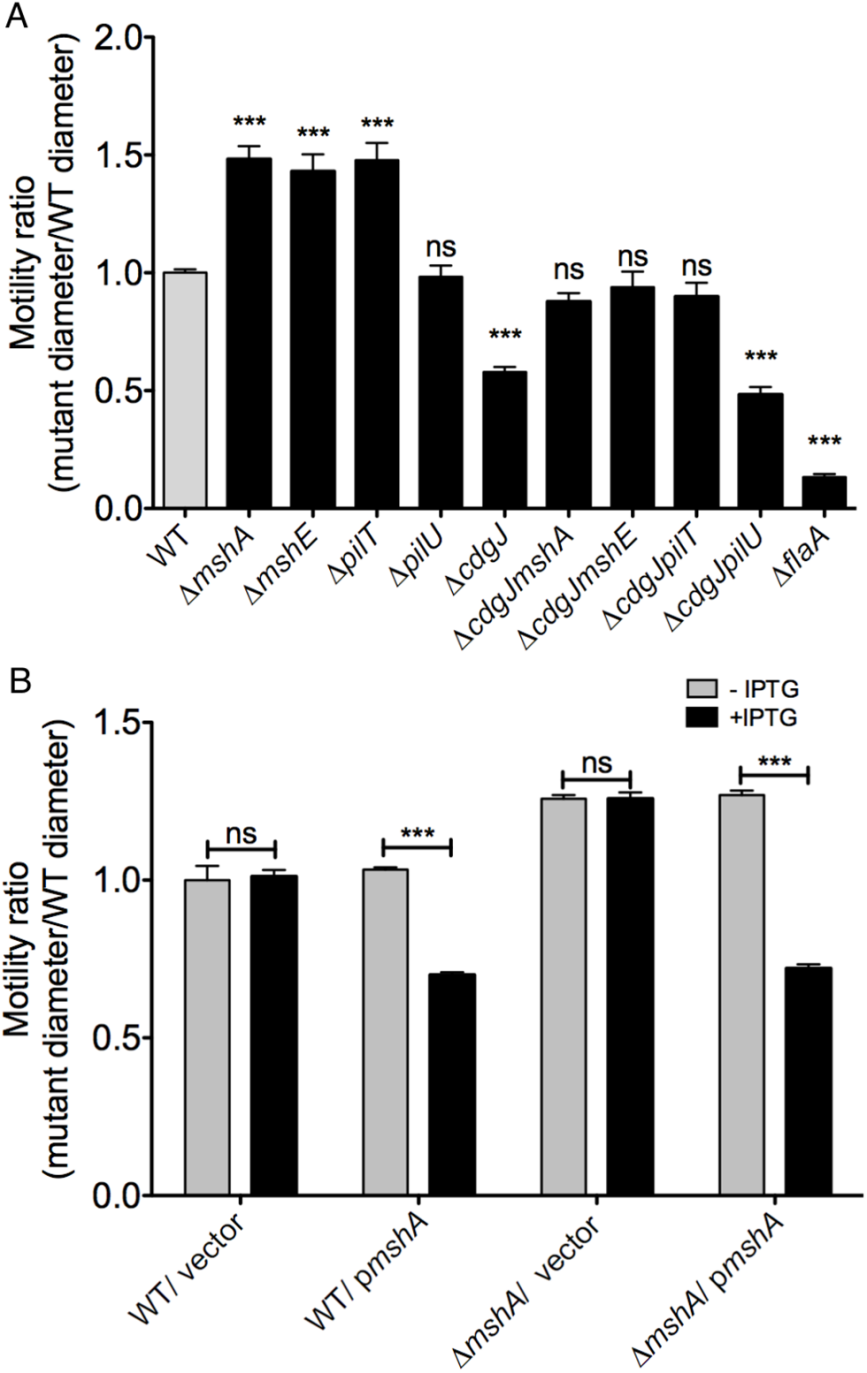
Mutations in *mshA*, *mshE*, and *pilT* increase motility. A. The diameters of migration zones of the WT and mutants were measured after 16 h of incubation at 30°C on LB soft agar motility plates and normalized to the motility of the WT strain. Four biological replicates were performed in quadruplicate. Statistical significance determined with Oneway ANOVA followed by Dunnett’s multiple comparison test comparing to the WT strain. (ns—not significant, *** p≤0.001) B. Expression of *mshA* reduces motility in soft agar. The diameters of migration zones were measured after 16 hours of growth at 30°C with or without IPTG induction and normalized to the motility of the WT strain. Two biological replicates were performed in triplicate. Induced strains compared to uninduced strains with Student’s t-test. (ns—not significant, *** p≤0.001).

To further investigate the suppression of a Δ*cdgJ* motility phenotype, we generated in-frame deletions of several of the genes identified in the transposon screen in wild-type and Δ*cdgJ* strains and analyzed the mutants for motility phenotype using LB soft agar motility assay. We focused on *mshA* (encoding major pilin subunit), *mshE* (encoding putative polymerizing ATPase), and *pilT* (encoding putative depolymerizing ATPase) as they are crucial for production and function of MshA pili. As previously reported, Δ*cdgJ* has a significant motility defect compared to the parental WT strain [36] (Fig 2A). Deletion of *mshA*, *mshE*, and *pilT* in a Δ*cdgJ* background restored motility similar to the WT strain, confirming that those mutations mediate suppression of the flagellar motility defect in the Δ*cdgJ* mutant. Deletion of *pilU* (predicted to encode a second copy of putative depolymerizing ATPase) in Δ*cdgJ* had no effect on the motility compared to the parental Δ*cdgJ* mutant. Mutants of *mshA*, *mshE*, and *pilT* in a WT background were assayed for motility to determine if their enhanced motility in the Δ*cdgJ* background is dependent on this mutation, or if this could be a case of bypass suppression. The Δ*mshA*, Δ*mshE*, and Δ*pilT* mutants exhibited enhanced motility compared to the WT strain (Fig 2A). The Δ*pilU* strain had a similar motility phenotype to the WT strain, suggesting that the functions or expression profiles of *pilT* and *pilU* are different. These data demonstrate that deletion of *mshA*, *mshE*, and *pilT* enhances motility regardless of the presence of a wild-type copy of *cdgJ*. However, we could not rule out the possibility that *cdgJ* could directly or indirectly control the production or function of the MshA pilus.

To determine if *mshA*-mediated suppression of the flagellar motility phenotype is specific to the *cdgJ* mutation or if it occurs in other PDE deletion backgrounds, we mutated *mshA* in a Δ*rocS* strain. RocS has both GGDEF and EAL domains and is predicted to function mainly as a PDE as Δ*rocS* mutants have reduced motility along with enhanced VPS production and biofilm formation [36–38]. We generated a Δ*rocSmshA* double mutant and determined that this mutant exhibited a wild-type motility phenotype (S1 Fig). These findings suggest that MshA negatively impacts *V*. *cholerae* flagellar motility and is involved in general c-di-GMP mediated repression of motility.

To evaluate further the ability of MshA to repress motility, we analyzed the effect of the expression of a WT copy of *mshA* provided in *trans* in an expression plasmid with an IPTG-inducible promoter. Motility assays confirmed that expression of *mshA*, upon induction with IPTG, significantly reduced motility in a Δ*mshA* strain (Fig 2B). Additionally, expression of *mshA* in the WT strain reduced motility, suggesting that overproduction of MshA impairs motility. IPTG had no effect on motility in strains harboring an empty vector control.

### *mshA*, *mshE*, and *pilT* mutants are deficient in biofilm formation

**Fig 3:**
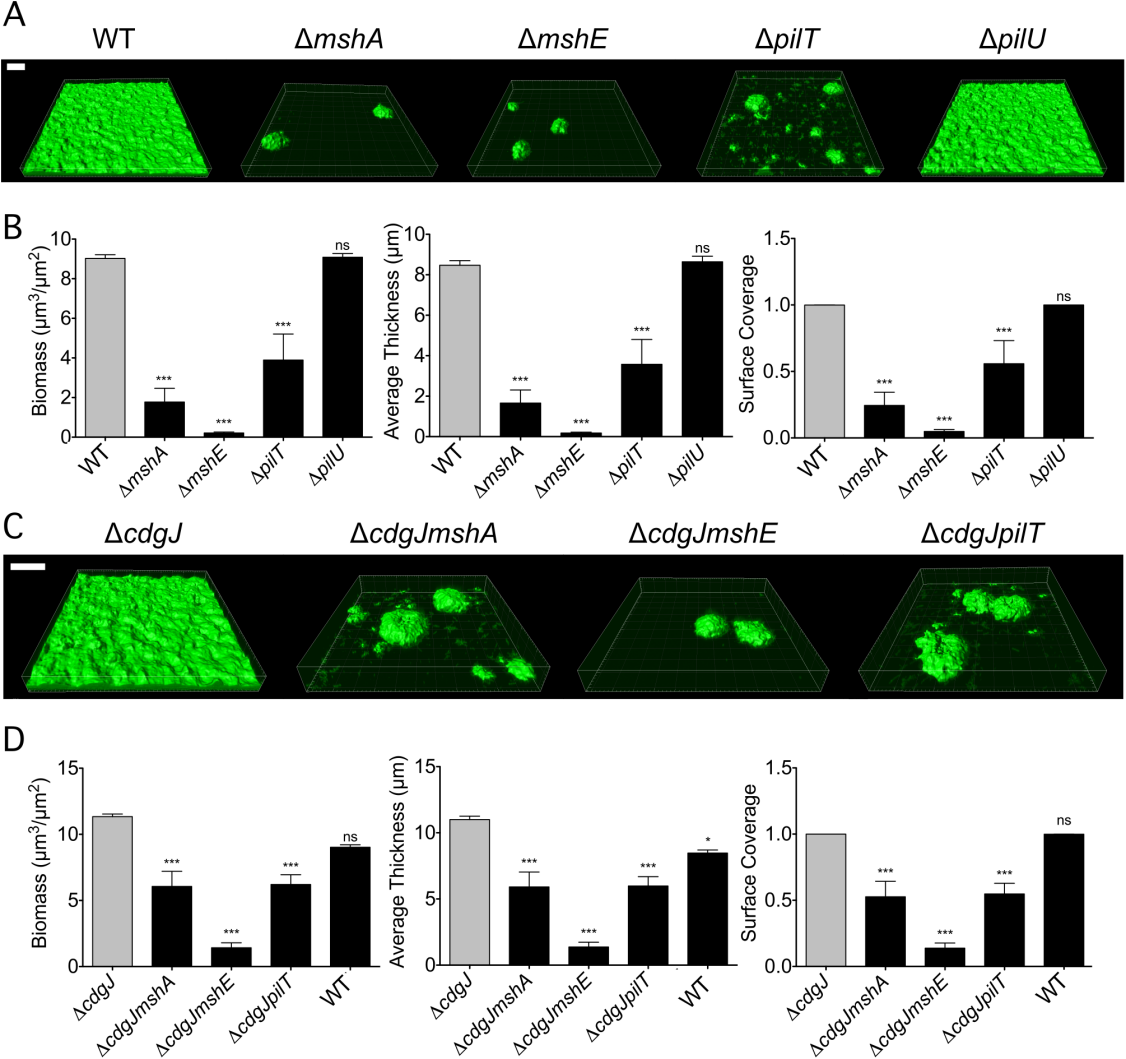
*mshA*, *mshE*, and *pilT* mutants are impaired in biofilm formation. A. Three-dimensional biofilm structures of WT, Δ*mshA*, Δ*mshE*, Δ*pilT*, and Δ*pilU* strains formed 24 h post inoculation in a flow cell system. Scale bar represents 20μm. B. COMSTAT quantitative analysis of biomass, average thickness, and surface coverage of biofilms from A. Three images from each of two independent experiments were analyzed. Significance was determined with a Oneway ANOVA followed by Dunnett’s Multiple Comparison test comparing to WT. (ns = not significant, *** p≤0.001) C. Three-dimensional biofilm structures of WT, Δ*cdgJ*, Δ*cdgJmshA*, Δ*cdgJmshE*, and Δ*cdgJpilT*, strains formed after 24 h post inoculation in a flow cell system. Scale bar represents 20μm. D. COMSTAT quantitative analysis of biomass, average thickness, and surface coverage of biofilms from C. Three images from each of two independent experiments were analyzed. Significance was determined with a Oneway ANOVA followed by Dunnett’s Multiple Comparison test comparing to Δ*cdgJ*. (ns = not significant, * p≤0.05, *** p≤0.001).

Since the MshA pilus is critical for initial stages of surface attachment and subsequent biofilm formation [10,11,20], we hypothesized that the Δ*mshE* and Δ*pilT* mutants would phenocopy the reduced biofilm phenotype of a Δ*mshA* mutant. Biofilms of these mutants were grown using a flow cell system, imaged using confocal microscopy, and analyzed using the COMSTAT image analysis software package to evaluate biofilm structural properties. As expected, the Δ*mshA* mutant attached poorly to the substrate and formed biofilms with low biomass (Fig 3A and 3B). The Δ*mshE* and Δ*pilT* mutants grew biofilms with significantly less biomass, thickness, and substrate coverage than WT. COMSTAT analysis revealed that while biofilm biomass of Δ*pilT* and Δ*mshA* was similar, the biomass of Δ*mshE* was significantly less than the Δ*pilT* strain (Fig 3B). Additionally, surface coverage of Δ*pilT* mutant was greater than that of Δ*mshA* and Δ*mshE*. These differences suggest that though the Δ*mshA*, Δ*mshE*, and Δ*pilT* mutants are deficient at forming biofilms, there are subtle differences in the phenotypes of the strains. The Δ*pilU* mutant produced biofilms that were indistinguishable from WT.

Since our initial interest in the MshA pilus was sparked by the discovery that mutations in pilus genes can suppress the motility defect in mutants of the PDE *cdgJ*, we determined whether the MshA pilus mutations were epistatic to the *cdgJ* mutation. As previously reported, the Δ*cdgJ* strain produced thicker biofilms than the WT strain (Fig 3C and 3D) [36]. The Δ*cdgJmshA*, Δ*cdgJmshE*, and Δ*cdgJpilT* strains formed biofilms with significantly less biomass, thickness, and surface coverage than the Δ*cdgJ* strain. These findings are consistent with the hypothesis that these proteins are involved in formation of the MshA pilus and that a functional MshA pilus is required for biofilm formation, surface attachment, and the inhibition of flagellar motility. Biofilms formed by the Δ*cdgJmshE* mutant had a significant reduction in biomass, thickness, and surface coverage compared to the Δ*cdgJmshA* or Δ*cdgJpilT* strains.

### *mshA*, *mshE*, and *pilT* mutants are defective in surface attachment and near surface motility

**Fig 4:**
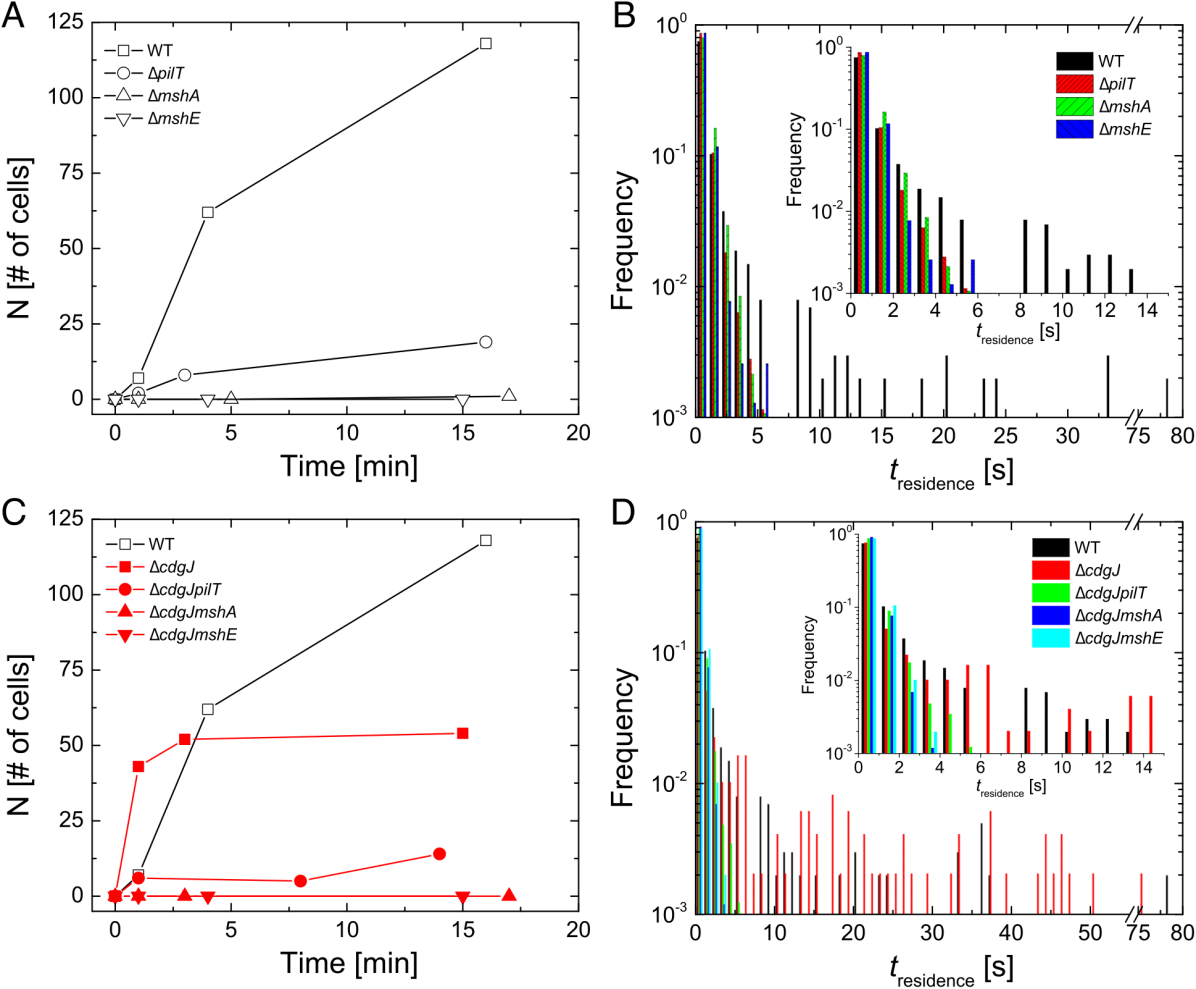
Early surface interactions are reduced in *mshA*, *mshE*, and *pilT* mutants and increased in *cdgJ* mutant. A, C. The number of surface-adhered, stationary cells were counted at the indicated times after inoculation in a flow cell. B, D. Residence times were measured for each cell that interacted with the surface during three high-speed movies of 82s in length within the first 15 minutes post inoculation.

In addition to using bulk differences in biofilm formation as an index of the transition between motile and sessile behavior, we also directly monitored surface attachment of *V*. *cholerae* with single cell resolution using high-speed microscopy and cell tracking (at 5 ms frame rate) to elucidate how genes involved in MshA pilus production impact microscopic outcomes such as initial surface attachment. Δ*mshA* mutants do not attach to the surface in significant numbers, with no attached cells observed in the first 15min after inoculation. In contrast, well over 100 cells of the WT strain attach to the surface over the same time interval (Fig 4A). Using the same metrics, the Δ*mshE* mutant was also unable to attach to the surface, exhibiting binding profiles that were similar to the Δ*mshA* strain. The Δ*pilT* strain was able to attach to the surface more than the Δ*mshA*; however, it is unable to attach with the same efficiency as the WT strain. This suggests the following hierarchy of behavioral categories for comparison with non-WT backgrounds: the strong binding strain (WT), the intermediate binding strain (Δ*pilT*), and the weak binding strains (Δ*mshA* and Δ*mshE*). To dissect the origin of this aggregate statistical behavior of surface attachment, we needed more single-cell metrics for bacterial behavior near a surface. We examined the temporal aspects of single bacterium interactions with the surface in the form of single cell residence time (number of seconds that each stationary cell remained associated with the surface, Fig 4B). The WT strain formed prolonged associations with the surface, with a mean residence time of 2.6 seconds and a maximum of 78.9 seconds. The Δ*mshA*, Δ*mshE*, and Δ*pilT* mutant strains demonstrated more transient interactions with the surface, with reduced mean residence times compared to WT (0.69, 0.58, and 0.58 seconds, respectively). This is most evident in the inset of Fig 4B, where the entire adherent populations of these mutants have residence times of less than 6 sec, while the WT residence times extend out to nearly 80 seconds.

### *mshA*, *mshE*, and *pilT* mutants are defective in near surface motility

**Fig 5:**
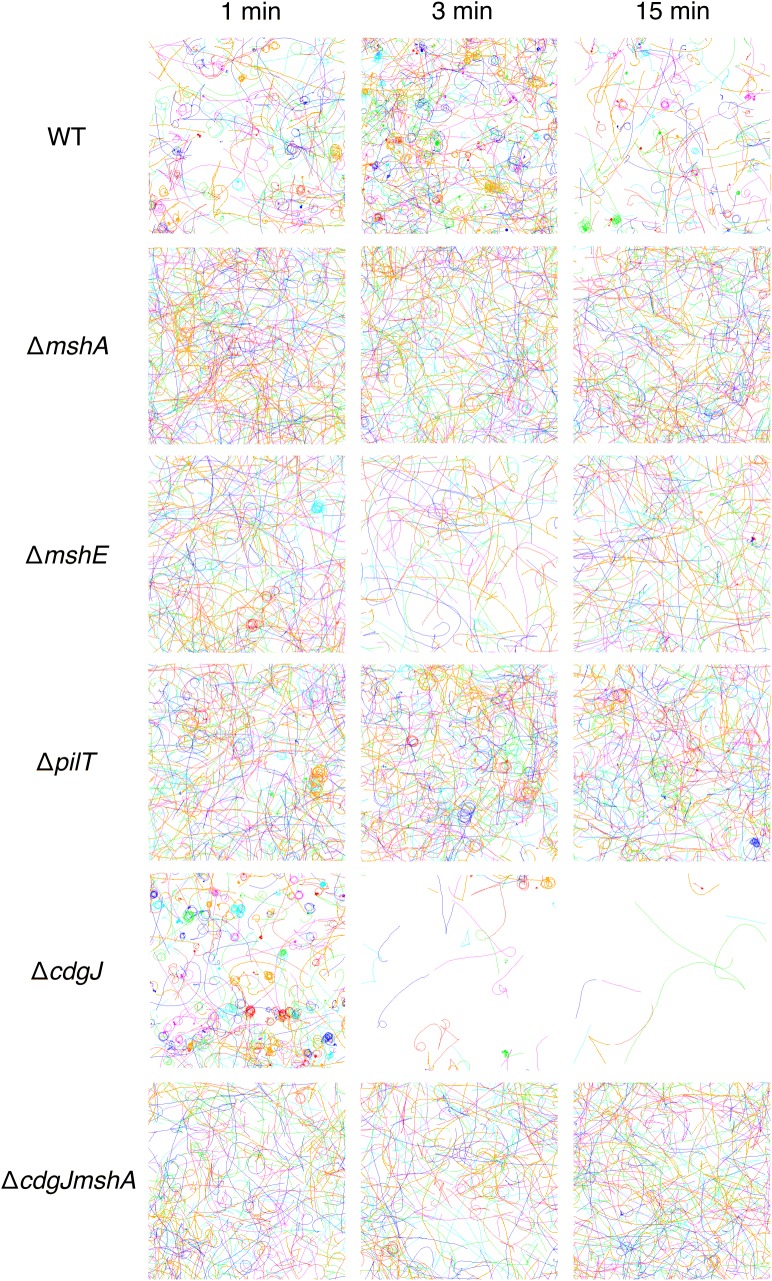
Mutations in *mshA*, *mshE*, *pilT*, and *cdgJ* affect near surface motility. Measured cell tracks at the indicated times post-inoculation. Bacterial populations were imaged for 82s with a 5 ms resolution, and a representative subset of individual cell tracks are presented for clarity. The field of view is 160μm x 160μm. Each line represents the motion of one cell and different colors are used to denote different cells. Non-motile cells have been filtered out of the data set at each time point for clarity.

We used high-speed microscopy and near-surface cell tracking to record the trajectories of cells within one micrometer of the coverslip surface in a microscopy chamber (Fig 5). All strains with flagella are capable of swimming motility in 3D, and can exhibit trajectories that come in and out of focus. Consistent with previous reports, the WT strain exhibits orbiting and roaming behavior with regards to near surface motility [10]. All of the surface attached cells come from the orbiting subpopulation. The Δ*mshA* mutant does not show orbiting or roaming behavior, consistent with the model that MshA pili-surface interactions are responsible for these near-surface motility phenotypes. Moreover, also consistent with the model, the mutant shows greatly reduced surface attachment (Fig 4A) [10]. The Δ*mshE* strain tracks phenocopy Δ*mshA*, exhibiting predominantly a swimming phenotype with little observable attachment. The Δ*pilT* mutant has an intermediate phenotype; a few cells appear to exhibit behavior similar to WT orbiting, but has greatly reduced attachment compared to WT. These data are consistent with the biofilm and attachment data described in Figs 2 and 3.

### *mshA*, *mshE* and *pilT* mutations are epistatic to a *cdgJ* mutation with regards to surface attachment

To investigate the role of the PDE CdgJ on initial surface attachment, we observed ***mshA*, *mshE*, *pilT*** mutants in a Δ*cdgJ* background with high-speed microscopy using similar experiments. We determined that the Δ*cdgJ* mutant exhibits strong attachment to the surface, with a sharp increase in the number of attached cells as a function of time during the initial few minutes compared to WT. (Fig 4C, time = 1). This strain rapidly associated with the surface, with nearly all cells binding within the first two minutes of observation. As predicted from the biofilm and motility data (Figs 1 and 2), the Δ*cdgJmshA*, Δ*cdgJmshE*, and Δ*cdgJpilT* strains exhibited reduced surface attachment compared to the parental Δ*cdgJ* strain and WT (Fig 4C). As in the WT background, the Δ*cdgJpilT* strain exhibited an intermediate level of attachment and the Δ*cdgJmshA* and Δ*cdgJmshE* strains were poor at attachment. The Δ*cdgJmshA*, Δ*cdgJmshE*, and Δ*cdgJpilT* strains exhibited short residence times with mean values of 0.43, 0.53, and 0.57 s, respectively (Fig 4D). By contrast, the Δ*cdgJ* strain exhibits much longer mean surface residence times (3.59 s with and a maximum of 75.8 seconds), which surpass even WT (Fig 4D, red vs black bars). These longer residence times indicate a strong tendency of the Δ*cdgJ* strain to associate with surfaces, which is also evident in the rapid decrease in density of tracks of the *ΔcdgJ* over time (Fig 5): since only motile cells are displayed in each image, the density of tracks diminishes over time in the *ΔcdgJ* strain due to the increasing proportion of adherent, nonmotile cells. These results demonstrate the Δ*cdgJ* strain adheres to surfaces more rapidly than WT, and that *mshA* is epistatic to *cdgJ*.

### Production of an extracellular MshA pilus is altered in Δ*mshE*, Δ*pilT*, and Δ*cdgJ*

**Fig 6:**
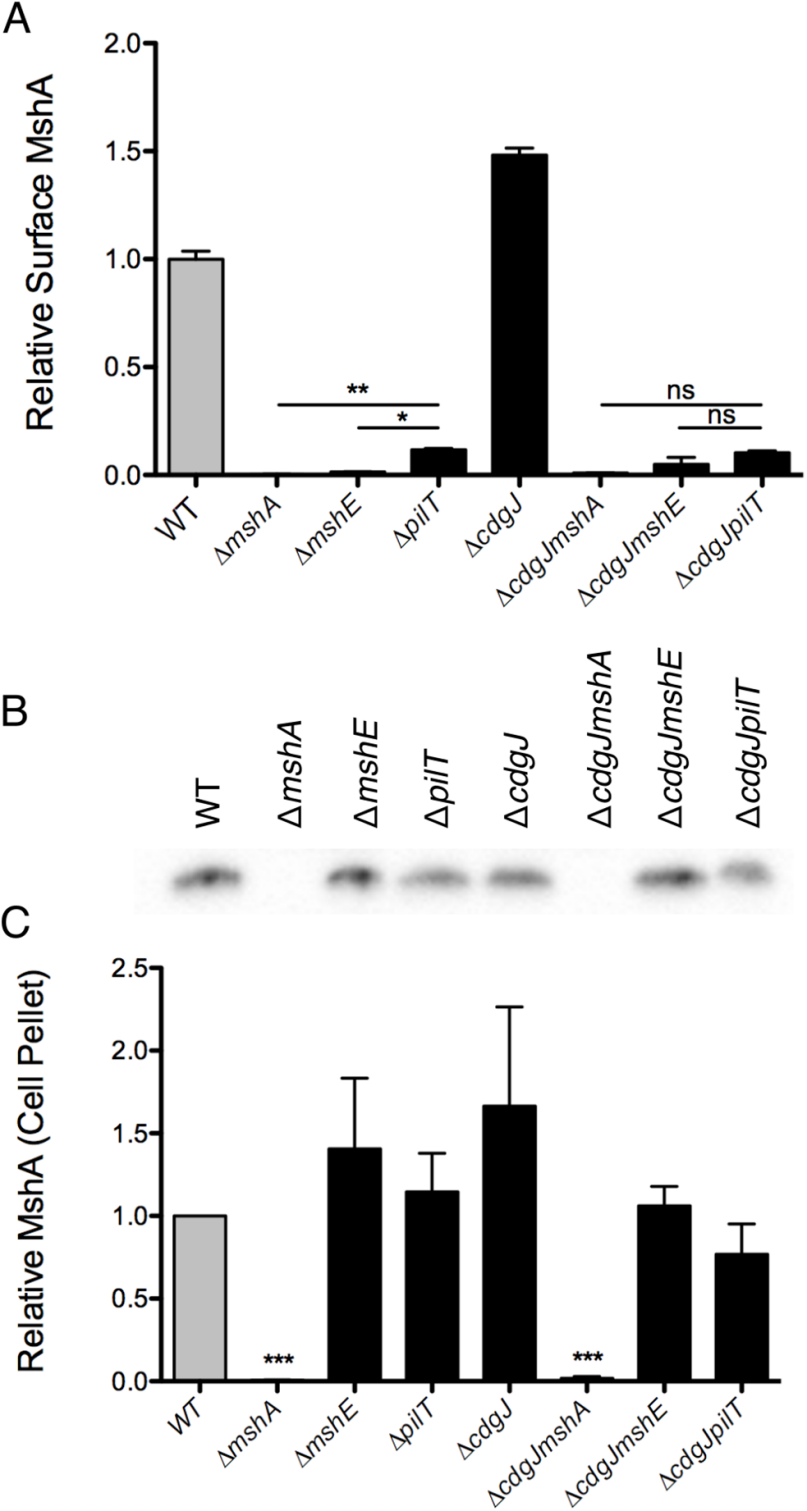
Production of surface MshA pili is MshE and PilT dependent and repressed by CdgJ. A. Surface MshA pilin production determined via ELISA with MshA-specific antibody. Two biological replicates were assayed in quadruplicate and normalized to the average of the WT strain. Oneway ANOVA followed by Bonferroni’s Multiple Comparison test. (ns = not significant, * p≤0.05, ** p≤0.01) B. Western blots detect MshA production from cell pellets using α-MshA antibody. Blots were performed in triplicate, with one representative image included. C. Densitometry of Western blots quantified MshA production in various strains. Densitometry analysis of blots was performed in triplicate and detection was normalized to the WT sample on each blot. Statistical significance determined using a one sample t-test to compare to WT. (*** p≤0.001).

The motility and biofilm phenotypes of the Δ*mshE* and Δ*pilT* mutants, combined with homology to known Type IV pilus motor proteins suggests that these genes are involved in the production of a functional MshA pilus. MshE shares 75% amino acid similarity with the Type IV extension ATPase PilB of *P*. *aeruginosa* and 77% similarity to the Type II extension ATPase EpsE of *V*. cholerae, suggesting that MshE is the extension ATPase of the MshA pilus (S2 and S3 Figs). We investigated the role of these genes in the production of MshA pili using a surface MshA pilin ELISA (Fig 6A), which detects only assembled pili. Δ*mshA*, Δ*mshE*, and Δ*pilT* mutants produced significantly less surface MshA pili than the WT strain. This supports the hypothesis that MshE and PilT are involved in the production of a functional MshA pilus. The Δ*pilT* strain produced significantly more surface pili than Δ*mshA* or Δ*mshE*, correlating with the intermediate phenotype of this strain observed in biofilm and near surface motility assays (Fig 3A and 3A). Surface MshA pilus production was determined in deletion mutants of other genes in the secretory operon with multiple transposon insertions (S4 Fig). Deletion of *mshL*, which encodes the putative outer membrane pore protein, resulted in no surface pili. While deletion of *mshM* (predicted to encode an ATPase) or *mshN* (predicted to encode a tetratricopeptide repeat domain) resulted in similar pilus production to the WT strain, pilus production was increased in a Δ*mshI* mutant, The specific mechanisms by which lack of these genes results in suppression of a motility defect in a Δ*cdgJ* strain is yet to be determined. The Δ*cdgJ* mutant produced significantly more surface MshA pili than WT. Surface MshA pili were reduced in Δ*cdgJmshA*, Δ*cdgJmshE*, and Δ*cdgJpilT* mutants to the level of the Δ*mshA* strain, indicating that none of these strains could produce a functional pilus. Whole cell western analysis indicated that all of the strains except Δ*mshA* and Δ*cdgJmshA* produced similar amounts of MshA protein, suggesting that the altered surface MshA is not a result of altered MshA production (Fig 6B and 6C). These data support the hypothesis that a functional MshA pilus inhibits motility and enhances surface attachment and biofilm formation. This effect is likely due to the interactions between the pili and surfaces, however, there may be additional mechanisms affecting motility and surface attachment, as discussed below. Additionally, the elevated production of MshA pili by the Δ*cdgJ* mutant could explain the motility and biofilm phenotypes observed in this mutant.

To confirm further that *mshE* was responsible for the lack of pili in a Δ*mshE* mutant, we generated chromosomal replacements at the native locus with either the WT *mshE* or a sequence encoding a mutation in the Walker A ATPase active site (K329A) (S5 Fig). Pilus production was restored to WT levels when the WT sequence was inserted, however the K329A produced no detectable surface pili. These data confirm that MshE, and specifically an intact ATPase domain, are required for MshA pilus production.

**Fig 7:**
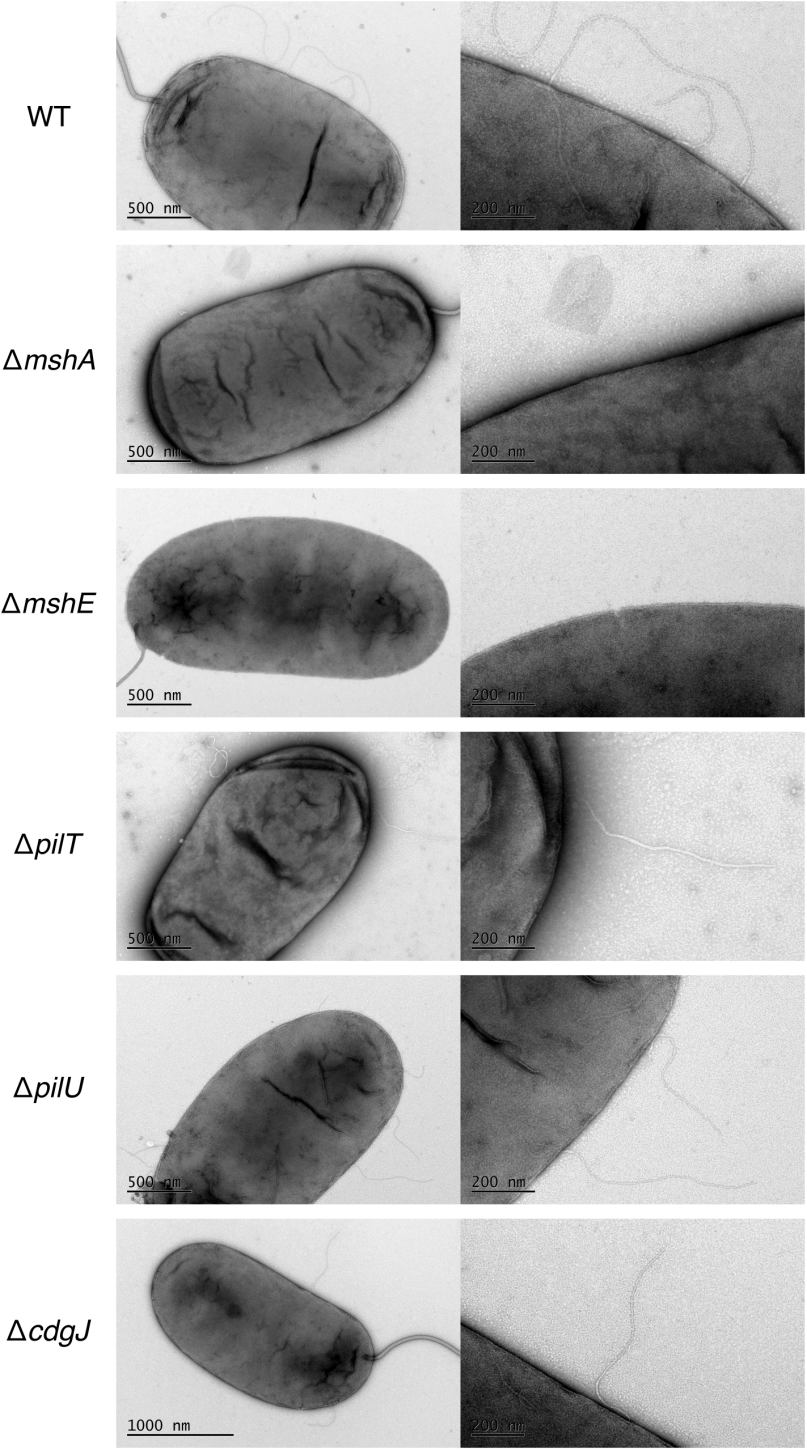
MshA pilus production is altered in an *mshE* mutant. Transmission Electron Microscopy of negative stained cells in exponential growth reveals MshA pili. Scale bars included for reference. Representative image presented of at least 8 images analyzed for each strain. Right panel is increased magnification to clearly visualize pili.

We also utilized transmission electron microscopy (TEM) to assess presence of MshA on the cell surface (Fig 7). We determined that WT produces several pili along the cell body (range 2–5) that were about one half to one cell body length. These pili were not present in the *mshA* mutant, suggesting that the pili observed are in fact MshA pili. Similarly, the Δ*mshE* strain produced no visible pili, which is consistent with the prediction that MshE is the motor protein responsible for extension of the MshA pilus. These images reveal that there were no differences observed between pili produced by WT and the Δ*pilT*, Δ*pilU* and Δ*cdgJ* mutants. Due to the fragile nature of MshA pili, quantitative measurements were not possible with TEM though these images observing the presence or absence of MshA pili support the quantitative measurements observed by ELISA.

### MshE binds c-di-GMP

**Fig 8:**
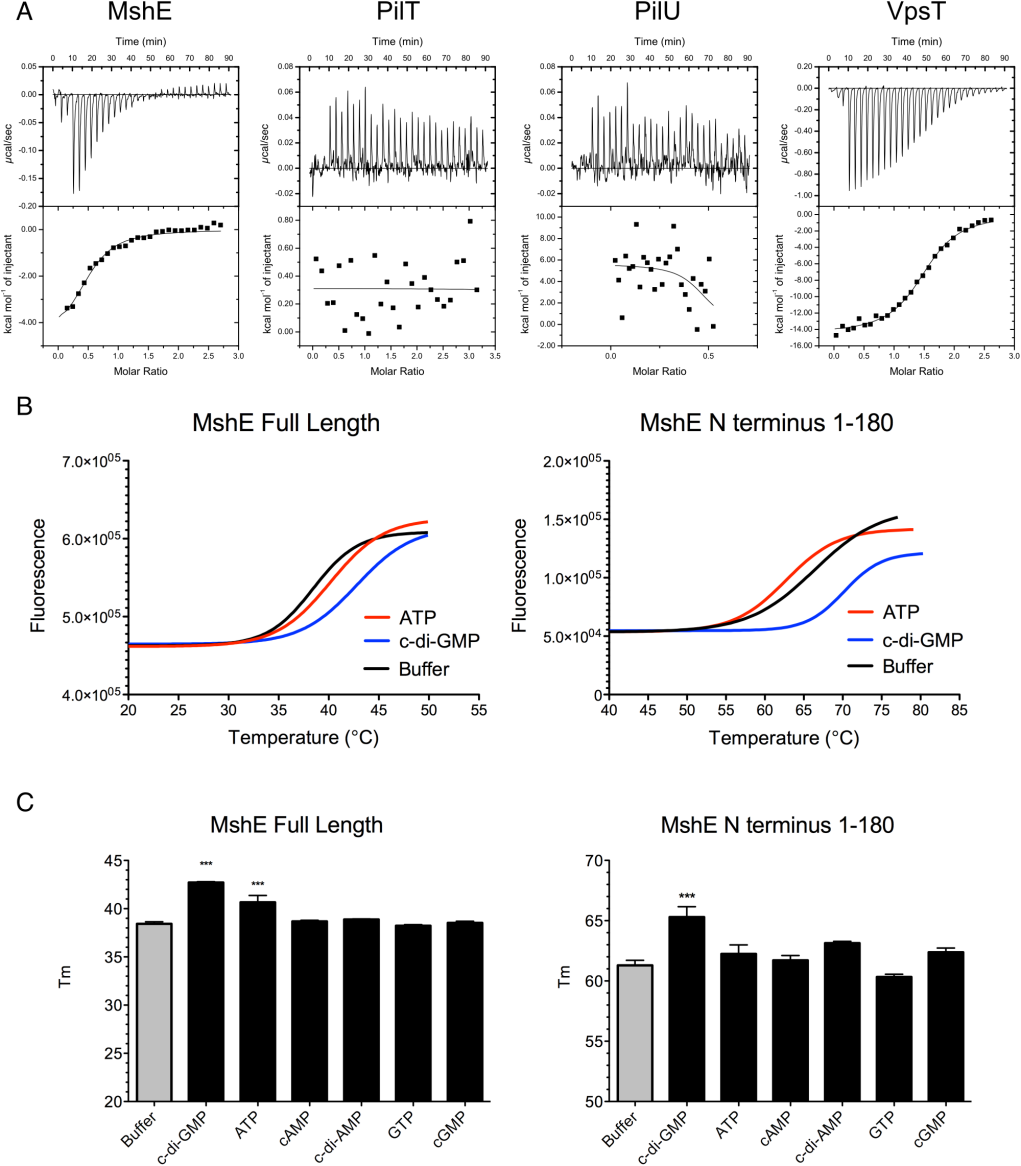
MshE binds c-di-GMP. A. Isothermal Calorimetry was performed on purified proteins to determine the binding affinity for c-di-GMP. Three independent runs were included in the analysis, one representative run is depicted. VpsT is included as a positive control for c-di-GMP binding. B. Fluorescence thermal shift was utilized to investigate domains of MshE responsible for binding c-di-GMP. Fluorescence melt curves are displayed in the presence of 2mM ATP or c-di-GMP. Three independent experiments were performed in triplicate, with one representative experiment shown. C. Midpoints of melt curves are displayed in the presence of 2mM nucleotides or a buffer control. Three independent experiments were performed in triplicate, Significance was determined with a Oneway ANOVA followed by Dunnett’s Multiple Comparison test comparing to Buffer control. (*** p≤0.001, all others not significant).

The pilus motor proteins MshE and PilT contain Walker A ATPase domains, which are utilized to energize the assembly and disassembly of pili. Baraquet *et al*. demonstrated that the activity of the *P*. *aeruginosa* regulator FleQ is modulated by binding c-di-GMP at its Walker A site [39]. We hypothesized that one or both of the Msh pilus motor proteins could function as a c-di-GMP receptor. Isothermal calorimetry was utilized to investigate the interaction of these proteins with c-di-GMP. We determined that MshE binds c-di-GMP, while PilT and PilU were unable to bind c-di-GMP (Fig 8A). ATPase activity of purified MshE, PilT, and PilU confirmed that these preparations contain functional protein (S6 Fig). VpsT was purified and included as a positive control, as it has been demonstrated to bind c-di-GMP [40]. Fitting the data to a single binding site model indicates that MshE has a slightly lower affinity for c-di-GMP (K = 1.14x10^5^ ± 3.24x10^4^ M^-1^) than VpsT (K = 9.9x10^4^ ± 2.06x10^4^ M^-1^). These data suggest that c-di-GMP does affect MshA pilus production through interactions with MshE.

Since MshE, PilT, and PilU contain conserved Walker A ATPase domains, but only MshE bound c-di-GMP, we purified the N terminal domain of MshE (amino acids 1–180) for analysis of the interactions with c-di-GMP. This domain lacks homology with either PilT or PilU and therefore is a likely candidate for binding c-di-GMP. Fluorescence thermal shift assays were utilized to determine the interaction between MshE or MshE-N terminal domain and nucleotide. Purified proteins are stabilized by a bound ligand; therefore the temperature midpoint of unfolding (T_m_) of the protein in the presence of ligand is higher than the T_m_ of the protein in buffer [41,42]. We observed that the T_m_ of full-length MshE in buffer was 38.43 ± 0.52°C (Fig 8B). When full-length MshE was incubated with ATP or c-di-GMP, the T_m_ increased to 40.15 ±1.02°C and 42.77±0.70°C, respectively. This indicates that full-length MshE binds both ATP and c-di-GMP. The N-terminal domain of MshE had a T_m_ of 66.36 ± 0.79°C in buffer. There was no increase in T_m_ in the presence of ATP (T_m_ = 62.72 ±0.46°C). In contrast, incubation of the MshE N-terminal domain with c-di-GMP increased the T_m_ to 70.18 ±0.55°C, indicating that this domain binds c-di-GMP (Fig 8C). Additionally, these interactions are specific, as neither the full-length nor the N-terminus of MshE bind to cAMP, c-di-AMP, GTP, or cGMP in thermal shift assays (Fig 8B and 8C).

### c-di-GMP promotes MshA pilus production

**Fig 9:**
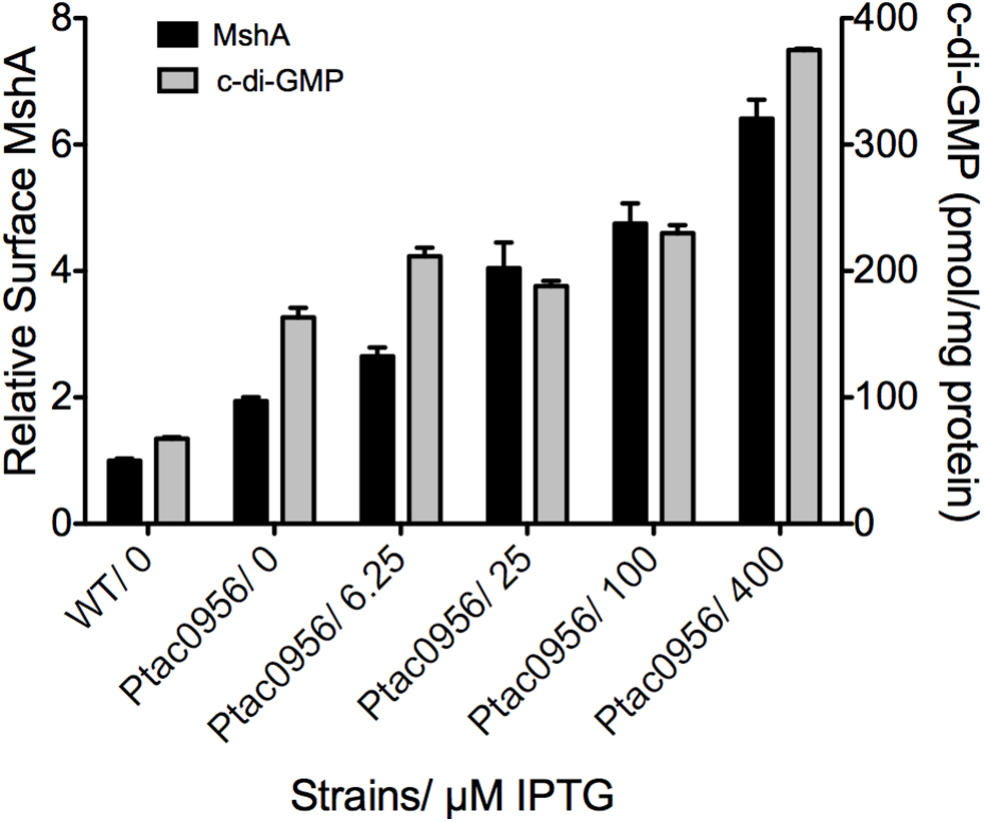
MshA pilus production correlates with c-di-GMP. Expression of the DGC VCA0956 was induced with varying amounts of IPTG. Intracellular c-di-GMP level was determined with LC-MS/MS and normalized to total protein in the extract (grey bars, right Y axis). Four independent samples were used for each induction condition. Oneway ANOVA followed by Dunnett’s Multiple Comparison Test determined significance. Surface MshA pilin was determined by ELISA (black bars, left Y axis). Three biological replicates were tested in triplicate. Results were normalized to MshA production in the WT strain. Surface MshA pili were significantly increased in all conditions compared to WT (Oneway ANOVA, Dunnett’s Multiple Comparison Test.) Also, there was a significant correlation between increased c-di-GMP and increased surface MshA pili (Pearson correlation, p = 0.0025, R^2^ = 0.9199).

To further evaluate the link between c-di-GMP production and pilus assembly, we performed a surface pilin ELISA to determine the production of MshA pili over a range of c-di-GMP concentrations (Fig 9). To generate a range of c-di-GMP concentrations, we introduced a construct harboring an IPTG-inducible copy of the DGC VCA0956 (Ptac0956) into the chromosome of the *V*. *cholerae* O1 El Tor strain A1552. This strain was grown in the presence of varying concentrations of IPTG, followed by detection of extracellular MshA with the surface pilin ELISA. We observed that upon induction of the expression of VCA0956 with IPTG, there was an increase in intracellular c-di-GMP (Fig 9, Grey bars). Additionally, the surface pilin ELISA detected increased amounts of extracellular MshA that coincided with the increased c-di-GMP (Fig 9, Black bars), though the total MshA production was not increased (S7B Fig) suggesting that the increased surface pilin is due to enhanced assembly. There was a significant correlation between elevated c-di-GMP and increased surface MshA (Pearson correlation, p = 0.0025, R^2^ = 0.9199). Increased c-di-GMP resulted in reduced motility and increased biofilm maximum thickness compared to WT (S7 Fig). Collectively, these data indicate that c-di-GMP promotes assembly of the MshA pilus. Induction of VCA0956 in a Δ*mshE* strain does not increase the production of surface MshA pili, further supporting that MshE is required for the c-di-GMP-dependent increase of MshA pili (S8 Fig).

## Discussion

High c-di-GMP levels inhibit motility of bacteria and studies have highlighted some of the mechanisms involved. c-di-GMP can repress transcription of flagellar genes, or can act post-transcriptionally to regulate flagellar reversals by interactions with particular flagellar motor proteins or by altering the chemotaxis signal transduction system [26–29,43–45]. Our work revealed a role for c-di-GMP in the regulation of MshA pilus production with effects on near surface motility, motile to sessile transition, and biofilm formation via a post-translational mechanism. c-di-GMP also promotes the assembly and activity of Type IV pili in *P*. *aeruginosa* [34,46]. In this example, ectopic expression of a DGC results in elevated amounts of c-di-GMP and increased surface pili. We demonstrate that production of the MshA pilus in *V*. *cholerae* is increased in response to high concentrations of c-di-GMP. These similar results in two organisms suggest that c-di-GMP may promote type IV pili assembly and activity as a more general mechanism of pilus regulation than previously identified. Many bacteria rely on type IV pili for motility and attachment, so integration of c-di-GMP in post-translational control of these structures could be a conserved mechanism across species.

The *V*. *cholerae* genome encodes 31 GGDEF domain proteins but we have found that only a subset of these impact motility, biofilm formation, or both. We previously reported that four DGCs CdgH, CdgK, CdgL, and CdgD and two PDEs CdgJ and RocS affect motility. Furthermore, a strain lacking all four DGC-encoding genes (Δ*cdgD*Δ*cdgH*Δ*cdgK*Δ*cdgL*) has a markedly high motility phenotype, suggesting the effect of these proteins on motility is additive [36]. Screening for suppressor mutants of Δ*cdgJ* that restored swimming motility identified CdgH and CdgK, suggesting that c-di-GMP produced by these DGCs could be a substrate for CdgJ. To test if CdgJ and DGCs that control motility physically interact, we analyzed interactions of CdgJ with CdgH, CdgK, CdgL, and CdgD using the commercially available Bacterial Adenylate Cyclase Two-Hybrid System (BACTH) (Euromedex Strasbourg, France). We did not observe any interaction between DGCs and CdgJ or between CdgJ and MshE or PilT, suggesting that these proteins do not need to be in physical contact, or the interaction is too weak or brief to be detected with this method.

Pili are dynamic structures that are generated by the assembly and disassembly of pilin subunits. The motor proteins responsible for this process have been characterized in many other systems. The two putative motor proteins of interest in this report, MshE and PilT, were identified based on homology to the PilT motor protein in *P*. *aeruginosa*. These proteins belong to AAA+ ATPase family proteins and are responsible for energizing the addition and disassembly of pilin subunits. Previous studies revealed presence of a bacterial AAA+ ATPase enhancer binding class of c-di-GMP receptors [28]. Here, we demonstrate that MshE binds to c-di-GMP. This is an important finding, as it establishes that ATPases beyond enhancer binding proteins are also capable of binding c-di-GMP. We note that PilT and PilU, which both harbor AAA+ ATPase domain, are unable to bind to c-di-GMP under the conditions tested. We also showed that N-terminal domain of MshE, which is not present in PilT and PilU is capable of binding to c-di-GMP. Future studies will elucidate the effect of c-di-GMP binding by MshE, as well as the specific mechanisms of this interaction.

We have also characterized several proteins necessary for the production of a functional MshA pilus. Although the function of members of the MshA operons were predicted based on homology to proteins of known function in other bacteria, function of these genes in MshA biogenesis were not characterized [22]. We have demonstrated that MshE is responsible for the assembly of MshA pilin subunits into a functional pilus. These data indicate that the decreased attachment and biofilm phenotypes, as well as the enhanced motility of the *mshA*, *mshE* and *pilT* mutants relies on the dynamic nature of the MshA pilus. If the presence of a pilus could enhance attachment and biofilm formation, a Δ*pilT* strain would in principle produce biofilms that had similar, or even greater biomass than the WT strain. The observation that the Δ*pilT* strain phenocopies Δ*mshA* and Δ*mshE*, which lack extracellular pili, suggests that both extension and retraction of the pili are critical for normal substrate attachment. The ELISA indicates that the Δ*pilT* strain produces fewer pili than WT, though the TEM images confirm that there are pili on the surface. Future studies will further investigate the role of PilT in the retraction of the MshA pilus. It is important to note that PilT has already been characterized as the retraction pilus of the ChiRP pilus (chitin-induced competence), suggesting that PilT can function in more than one type IV pilus system [47]. This could be a mechanism for genomic conservation, where one promiscuous retraction ATPase is encoded, while several extension ATPases allow for specificity of the system in regulation. Future studies will address the mechanism of co-regulation of these systems to determine whether *pilT* is expressed constitutively while the specific extension ATPases are regulated, or if there is overlap in the regulation between the extension and retraction ATPases.

Several studies have investigated how bacteria sense and respond to surfaces, often times by rapidly upregulating production of adhesins and polysaccharides [48–50]. This regulation is typically mediated by c-di-GMP [23,28,40,50,51]. Many bacteria also regulate motility in response to surfaces. *E*. *coli* uses the resistance to flagellar rotation as a mechanosensor and adapt by adding force-generating motor subunits to the stator complex [43]. This allows the bacterium to adjust the force of flagellar rotation to match the viscosity of the environment. An additional example of “stator swapping” to modulate flagellar force was recently published for *P*. *aeruginosa* [44]. In this example, c-di-GMP represses motility by excluding the swarming proficient MotC/D proteins from the stator complex in favor of the swarming deficient MotA/B proteins. *P*. *aeruginosa* also utilizes the altered chemotaxis protein WspA as a surface sensor, which results in production of c-di-GMP by the DGC WspR upon interaction with a surface [52,53]. In *B*. *subtilis*, production of flagella, and therefore swarming motility, is regulated by Lon-dependent proteolysis of the master regulator of flagellar biosynthesis SwrA upon surface contact [54]; and flagellar function is modulated by EpsE which synergizes exopolysaccharide biosynthesis with flagellar motility by acting as a clutch through interaction with the flagellar protein FliG to limit rotation and therefore motility [55].

This study presents a possible mechanism for how c-di-GMP production affects motility and biofilm formation through modulating MshA pilus production. Both pili and flagella contribute to near surface motility and initial attachment [10]. Besides the generation of near-surface motility modes conducive to surface attachment, it is known that van der Waals forces depend crucially on the extent of surface contact [56]. That *V*. *cholerae* has a comma-like helicoid shape with smaller surface contact areas implies that adhesive forces between the surface and the cell body will be decreased relative to more cylindrically symmetrical species such as *P*. *aeruginosa* for most cell orientations, so adhesive contributions from appendages like MSHA to ‘anchor’ the cell on surface will be comparatively more important. In fact, recent measurements of TFP adhesive forces show that they can be quite strong, in the hundreds of pico-Newton (pN) range, and are surface chemistry dependent, in agreement with our results [57]. That *V*. *cholera* select for surfaces that interact with MSHA strongly (and thereby generate ‘orbiting’ motility) implies that the existence of more functional MSHA induced by c-di-GMP can better anchor a cell mechanically and mitigate against flagellum driven motion. This work further strengthens the notion that there is a mechanistic link between c-di-GMP and initial attachment through modulation of flagellar motility and pilus activity.

## Materials and Methods

### Bacterial strains, plasmids, and culture conditions

The bacterial strains and plasmids used in this study are listed in S1 Table. All *V*. *cholerae* and *Escherichia coli* strains were grown aerobically, at 30°C and 37°C, respectively, unless otherwise noted. All cultures were grown in Luria-Bertani (LB) broth (1% Tryptone, 0.5% Yeast Extract, 1% NaCl), pH 7.5. LB agar medium contains 1.5% (wt/vol) granulated agar (BD, Sparks, MD). Concentrations of antibiotics and inducers used, where appropriate, were as follows: ampicillin, 100 μg/ml; rifampicin, 100 μg/ml; gentamicin, 50 μg/ml, kanamycin, 50 μg/ml, and arabinose, 0.2% (w/v), 6.25–400μM IPTG. In-frame deletion and GFP-tagged strains were generated according to protocols previously published [13,14].

### Recombinant DNA techniques

DNA manipulations were carried out by standard molecular techniques according to manufacturer’s instructions. Restriction and DNA modification enzymes were purchased from New England Biolabs (Ipswitch, MA). Polymerase chain reactions (PCR) were carried out using primers purchased from Bioneer Corporation (Alameda, CA) and the Phusion High-Fidelity PCR kit (New England Biolabs, Ipswitch, MA), unless otherwise noted. Sequences of the primers used in the present study are available upon request. Sequences of constructs were verified by DNA sequencing (UC Berkeley DNA Sequencing Facility, Berkeley, CA).

### Generation of a c-di-GMP overproducing strain

A region encompassing the P_lacIq-_ *lacI* and the P_tac_ promoter elements was amplified from the pMAL-c5x plasmid (New England Biolabs, Ipswitch, MA) by PCR. The amplified product was joined by overlapping PCR to amplicons of ~500 bp that correspond to sequences upstream and downstream of the VCA0956 translational start site. The resulting amplicon was cloned into the suicide plasmid pGP704*sacB*28 and mobilized into *Vibrio cholerae* A1552 by biparental mating. The selection of double recombinants with the desired insertion of the P_lacIq-_ *lacI* and P_tac_ promoter elements was performed as described in [14]. Sequences of constructs were verified by DNA sequencing (UC Berkeley DNA Sequencing Facility, Berkeley, CA).

### Transposon mutagenesis

To generate a library of transposon mutants, *V*. *cholerae* O1 El Tor strain A1552 *ΔcdgJ* was conjugated with the donor *E*. *coli* S-17-l λpir containing the Mariner transposon on the pSC189 backbone [58]. Transconjugants were selected on LB agar containing kanamycin 50μg/ml and rifampicin 100μg/ml. A total of 7054 mutants were isolated and screened for motility phenotypes on LB soft agar (0.3%) motility plates.

### Plate motility assay

Motility plates consist of LB containing 0.3% agar supplemented with 100μM IPTG where appropriate. Plates were poured and allowed to dry at room temperature for 4 h prior to inoculation. Colonies from overnight LB agar plates grown at 30°C were transferred to motility plates and incubated for 16 h at 30°C. Motility diameter was measured and normalized to the average of WT on each plate. Experiments were performed with three biological replicates in triplicate and data were analyzed with a Oneway ANOVA followed by Dunnett’s multiple comparison test.

### Confocal laser scanning microscopy (CLSM) and flow cell biofilm studies

Inoculation of flow cells was done by diluting overnight-grown cultures to an OD600 of 0.04 and injecting into a μ-Slide VI0.4 (Ibidi, Martinsried, Germany). To inoculate the flow cell surface, bacteria were allowed to adhere at room temperature for 1 h. Flow of 2% v/v LB (0.02% tryptone, 0.01% yeast extract, 1% NaCl; pH 7.5) was initiated at a rate of 7.5 ml/h and continued for 24 h. Confocal images were obtained on a Zeiss LSM 5 PASCAL Laser Scanning Confocal microscope. Images were obtained with a 40X dry objective and were processed using Imaris (Bitplane, Zurich, Switzerland). Quantitative analyses were performed using the COMSTAT software package [59]. Statistical significance was determined using Oneway ANOVA with Dunnett’s Multiple Comparison test. Three biological replicates were performed in triplicate. Images presented are from one representative experiment.

### High-speed imaging and cell tracking

Bacteria were cultured in full strength Luria–Bertani (LB) broth overnight under shaking at 30°C. Immediately prior to inoculation, cultures were diluted into 2% LB (containing 171 mM NaCl) to an OD600 0.01–0.03. The *V*. *cholerae* cells were then injected into a sterile flow-cell containing the same media and imaged immediately.

Imaging was performed with a Phantom V12.1 high-speed camera (Vision Research) collecting ~20,000 bright-field images at 5 ms resolution with a 100x oil objective on an IX71 Olympus microscope. All movies were recorded at the same frame rate, for the same duration, after the first, third and 15th minute post inoculation.

For cell-tracking algorithms and analysis protocol, every frame of a movie was preprocessed in Matlab (Mathworks) by subtracting the background, scaling, smoothing and thresholding. Image processing this way causes the bacteria appear as bright regions. Tracking is done by locating all bright objects that overlap objects in the next frame by combining the two frames into a three-dimensional (3D) matrix and then by locating 3D connected components.

Results are stored in a tree-like data structure with multiple roots; every newly detected bacterium that appears is recorded as a ‘root’ of the tree. When bacteria interact, they are recorded as a ‘node’ of the tree; when they depart, they are recorded as a ‘leaf’. Each root or node stores the sequence of pixel lists that comprise the bacterium in all frames until the next interaction or detachment event. We measure the instantaneous shape properties of the bacteria using the Matlab regionprops function [10].

### Surface Pilin ELISA

Surface pili composed of MshA were quantified using an ELISA based on a previously published protocol [46]. Briefly, overnight culture was diluted 1:100 in fresh LB medium and grown to OD_600_ 0.5 at 30°C. Cells (125μL) were added to a 96-well plate (Greiner Bio-One, Monroe, NC) and incubated at 30°C for one hour. Cells were fixed with 100μL of methanol for 10 minutes at room temperature, then washed twice with PBS. Samples were blocked in 5% nonfat dry milk and immunoblotted with polyclonal rabbit anti-MshA (1:1000 dilution, gift of J. Zhu) and horseradish peroxidase (HRP)-conjugated secondary antibody (Santa Cruz Biotechnology, Santa Cruz, CA). After three washes in PBS, 100μL of TMB (eBioscience, San Diego, CA) was added and incubated for 30 minutes at room temperature followed by the addition of 100μL of 2N H_2_SO_4_. Absorbance was recorded at 490nm and the samples were normalized to the change in WT. Three biological replicates were assayed in triplicate and statistical significance was determined with a Oneway ANOVA followed by a Dunnett’s Multiple Comparison test.

### Western blot

Samples were grown to mid-exponential phase (OD_600_0.5) in LB or LB with IPTG. Cells were collected via centrifugation and cell pellets were resuspended in 2% SDS and boiled for 5 minutes. Lysates were cleared via centrifugation and total protein was quantified via BCA assay (Pierce, Rockford, IL). Two hundred μg of protein was separated on a 12% SDS PAGE gel and transferred to a PVDF membrane using a semi-dry transfer apparatus (Bio-Rad, Hercules, CA). Blots were blocked in 5% nonfat dry milk and immunoblotted with polyclonal rabbit anti-MshA (1:2000 dilution, gift of J. Zhu) and horseradish peroxidase (HRP)-conjugated secondary antibody (Santa Cruz Biotechnology, Santa Cruz, CA). Chemiluminescence was detected with the SuperSignal West Pico reagents (Pierce, Rockford, IL) on the ChemiDoc MP Imager (Bio-Rad Hercules, CA). Densitometry was performed using the Image Lab software v4.0.1 (Bio-Rad, Hercules, CA) using the band in the WT lane as a reference. Blots were performed in triplicate for densitometry analysis and a representative image is shown.

### Transmission Electron Microscopy

Bacteria were prepared for electron microscopy by inoculating a single colony of each *V*. *cholerae* strain in LB broth grown overnight at 30°C with shaking at 200 rpm after which each culture was diluted 1:100 in LB broth and allowed to grow similarly to OD 0.4. An aliquot of each culture was diluted to yield an optical density of 0.1–0.2 and then applied to a 300 mesh carbon-coated Formvar grid (Electron Microscopy Sciences, Hatfield, PA). After 2 minutes, each grid was washed five times with deionized water, and negatively stained with 2% (w/v) aqueous uranyl acetate solution for 90 seconds. Imaging was performed with a JEOL JEM-1400 transmission microscope.

### Protein purification

*E*. *coli* BL21 harboring plasmids for gene expression were grown to an OD_600_ of 0.4 at 30°C in LB containing 100μg/mL ampicillin. Cultures were shifted to 18°C and IPTG was added to a final concentration of 100μM. 16h post induction, cells were harvested by centrifugation at 10,000 x g for 15 minutes and stored at -80°C.

Cell pellets were resuspended in GST Lysis Buffer (50mM Tris (pH 8.0), 1M NaCl, 0.5% Tween-20 containing PI cocktail tablets (Roche Life Science, Indianapolis, IN). Cells were lysed by sonication and cell lysate was cleared via centrifugation. Cleared lysate was loaded onto GST FPLC column as follows.

GSTPrep FF16/10 column (GE Healthcare, Piscataway, NJ) was equilibrated in lysis buffer at 1mL/minute using a BioLogic DuoFlow FPLC system (Bio-Rad, Hurcules, CA). Sample was loaded and washed with 1 column volume of GST Lysis Buffer (20mL). Subsequent washes were performed with 20mL of Wash Buffer 2 (50mM Tris (pH 8.0), 0.25M NaCl, 0.5% Tween-20, 0.5mM DTT) and 3 (50mM Tris (pH 8.0), 0.25M NaCl, 0.5mM DTT). Bound protein was eluted with 80mL of GST Elution Buffer 4 (50mM Tris (pH 8.0), 0.25M NaCl, Glutathione 1.5g/L) and collected in 15mL fractions.

These fractions were pooled and concentrated to approximately 10mL using an Amicon 10KDa cutoff spin fliter (EMD Millipore, Darmstadt, Germany). Samples were dialyzed against ITC buffer (25mM Tris-HCl, 150mM NaCl, 250μM DTT, pH 7.5) overnight using 12 kDa cutoff dialysis tubing (Fisherbrand, Pittsburgh, PA). An aliquot of dialyzed protein was diluted in 6M guanidinium HCl and concentration determined via A_280._

### Isothermal calorimetry

MshE (18.9μM), PilT (20.2μM), PilU (21.1μM), VpsT (19.5μM) and c-di-GMP (250μM) were prepared in 25mM TrisHCl pH 7.5, 150mM NaCl, and 200μM DTT and degassed prior to analysis. ITC was performed in with a VP-ITC (MicroCal, Northampton, MA) with the following parameters: 3 initial injections of 2μL followed by 40 injections of 10μL spaced at 180 seconds. The data were normalized to a run injecting c-di-GMP into buffer to account for the heat of dilution. Data were processed in Origin v7.0 software (OriginLab, Northampton, MA) and fit to a single site model.

### Fluorescence thermal shift

Thermal shift assays were performed as previously described with modifications [41,42]. Briefly, purified protein was added to the reaction to a final concentration of 3μM in the presence or absence of 2mM concentration of the indicated nucleotide in buffer (25Mm TrisHCl pH 7.5, 100mM NaCl, 1:1000 dilution of SYPRO Orange Dye (Invitrogen), and 0.2mM MgCl_2_. A melt curve protocol was run on an Applied Biosystems ViiA7 qPCR instrument. The fluorescence was measured using the ROX reporter with a temperature gradient of 20–95°C in 0.5°C increments at 30 second intervals. Melt curve data were trimmed to three data points after maximum and the data were plotted with Boltzmann model to obtain the temperature midpoint of unfolding (T_m_) of the protein in each condition using Prism 5.0 software (GraphPad). The fluorescence baseline of each sample was normalized to the buffer control for visualization purposes. Three biological replicates were assayed in triplicate and statistical significance was determined with a Oneway ANOVA followed by a Dunnett’s Multiple Comparison test.

### ATPase assay

ATPase activity of purified proteins was determined by measuring the production of inorganic phosphate from ATP using the Enzchek Phosphate Assay Kit (Invitrogen). The standard reaction mixture was prepared with the addition of 2mM MgCl_2_, 10mM KCl, and 1mM DTT. Purified protein in buffer (25mM TrisHCl pH 7.5, 100mM NaCl) was added to the standard reaction mixture to a final concentration of 5μM. After a 10 minute incubation at room temperature, ATP was added to a final concentration of 10mM and reactions were incubated at 22°C for 30 minutes. Production of inorganic phosphate was monitored by reading OD_360_ and compared to a standard curve of solutions of KH_2_PO_4_. The data are reported as specific activity (nmol Pi/min/mg of protein). BSA was included as a negative control. Three independent experiments were run in triplicate.

### c-di-GMP measurement

c-di-GMP extraction was performed as described previously [36]. Briefly, 40 ml of culture grown to OD_600_ ~0.4 was centrifuged at 3220 x *g* for 30 min. Cell pellets were allowed to dry briefly then re-suspended in 1 ml extraction solution (40% acetonitrile, 40% methanol, 0.1% formic acid, 19.9% water), and incubated on ice for 5 min. Samples were then centrifuged at 16,100 *g* for 5 min and 800 μl of supernatant was dried under vacuum and lyophilized. Samples were re-suspended in 50 μl of 184 mM NaCl and analyzed by liquid chromatography-tandem mass spectrometry (LC-MS/MS) on a Thermo-Electron Finnigan LTQ mass spectrometer coupled to a surveyor HPLC (Thermo, Waltham, MA). The Synergin Hydro 4u Fusion-RP 80A column (150 mm x 2.00 mm diameter; 4-μm particle size) (Phenomenex, Torrance, CA) was used for reverse-phase liquid chromatography. Solvent A was 0.1% acetic acid in 10 mM ammonium acetate, solvent B was 0.1% formic acid in methanol. The gradient used was as follows: time (t) = 0–4 min, 98% solvent A, 2% solvent B; t = 10–15 minutes, 5% solvent A, 95% solvent B. The injection volume was 20 μl and the flow rate for chromatography was 200 μl/minutes.

The amount of c-di-GMP in samples was calculated with a standard curve generated from pure c-di-GMP suspended in 184 mM NaCl (Biolog Life Science Institute, Bremen, Germany). Concentrations used for standard curve generation were 50 nM, 100 nM, 500 nM, 2 μM, 3.5 μM, 5 μM, 7.5 μM, and 10 μM. The assay is linear from 50 nM to 10 μM with an R^2^ of 0.999. c-di-GMP levels were normalized to total protein per ml of culture.

To determine protein concentration, 4 ml from each culture was pelleted, the supernatant was removed, and cells were lysed in 1 ml of 2% sodium dodecyl sulfate. Total protein in the samples was estimated with BCA assay (Pierce, Rockford, IL) using bovine serum albumin (BSA) as standards. Each c-di-GMP quantification experiment was performed with four biological replicates. Levels of c-di-GMP were compared to WT with Oneway ANOVA followed by a Dunnett’s Multiple Comparison test.

## Supporting Information

S1 Fig Motility of Δ*rocS* is restored to WT by deletion of *mshA*. The diameters of migration zones of the WT and mutants were measured after 16 h of incubation at 30°C on LB soft agar motility plates and normalized to the motility of the WT strain. Three biological replicates were performed in quadruplicate. Statistical significance determined with Oneway ANOVA followed by Dunnett’s multiple comparison test comparing to the WT strain. (ns—not significant, *** p≤0.001)
(TIFF)

S2 Fig MshE is homologous to PilB of *Pseudomonas aeruginosa*. Amino acid alignment of MshE from *V*. *cholerae* and PilB from *Pseudomonas aeruginosa* indicates that there is 35% identity and 75% similarity. Stars indicate amino acid identity, colons indicate high similarity, and periods indicate similar amino acids. The conserved WalkerA, Asp Box, Walker B, and His Box domains are shaded.
(TIFF)

S3 Fig MshE and PilT are homologous to the Type II secretion ATPase EpsE. Amino acid alignment of MshE and EpsE indicates that there is 37% identity and 77% similarity, while PilT and EpsE share 26% identity and 73% similarity. Stars indicate amino acid identity, colons indicate high similarity, and periods indicate similar amino acids. The conserved WalkerA, Asp Box, Walker B, and His Box domains are shaded.
(TIFF)

S4 Fig Surface MshA pilus production is dependent on MshA, MshE, and MshL. Strains with clean deletions of genes with multiple deletions in the secretory operon were generated and assayed for surface MshA pilus production. Deletion of *mshA*, *mshE*, and *mshL* abrogates production of surface MshA pili, while deletion of *mshI*, *mshM*, and *mshN* did not decrease MshA pilus production. Two biological replicates were assayed in quadruplicate and normalized to the average of the WT strain. Oneway ANOVA followed by Dunnett’s Multiple Comparison test compared to WT. (* p≤0.05, ** p≤0.01)
(TIFF)

S5 Fig MshE K329 is required for MshA pilus production. Surface pilin ELISAs indicate that replacing the chromosomal copy of *mshE* with a mutant of the lysine in the Walker A domain (K329A) abrogates pilus production. Three independent experiments were performed in triplicate. Significance was determined with an Oneway ANOVA followed by Dunnett’s Multiple Comparison Test compared to WT. (*** p≤0.001, all others not significant)
(TIFF)

S6 Fig ATPase activity of purified proteins. Production of inorganic phosphate from ATP by purified protein preparations was observed to determine functionality of purified proteins. The specific activity of a 5μM solution of protein with and without ATP is displayed after 30 minutes of incubation. Bovine Serum Albumin (BSA) was included as a negative control. Three independent experiments were performed in triplicate.
(TIFF)

S7 Fig Motility and biofilm phenotypes of Ptac0956 strain. A. The diameter of migration zone of the WT and Ptac0956 strain were measured after 16 h of incubation at 30°C on LB soft agar motility plates containing a range of IPTG and normalized to the motility of the WT strain. Three biological replicates were performed in quadruplicate. B. Western blots detect MshA production over a range of IPTG concentrations (0, 6.25, 25, 100, 400μM) from cell pellets using α-MshA antibody. Blots were performed in triplicate, with one representative image included. C. Three-dimensional biofilm structures of the *V*. *cholerae* strains formed 24 h post inoculation in a flow cell system. Scale bar represents 40μm. Comstat analysis of two independent experiments in triplicate indicated that Ptac0956 with 400μM IPTG had significantly increased maximum thickness compared to WT (WT 13.42 ±1.50μm, Ptac0956 17.60±1.25μm p≤0.01).
(TIFF)

S8 Fig MshA pilus production is not affected by increased c-di-GMP in a Δ*mshE* strain. Expression of the DGC VCA0956 was induced with varying amounts of IPTG. Surface MshA pilin was determined by ELISA. Three biological replicates were tested in triplicate. Results were normalized to MshA production in the WT strain. Surface MshA pili production in the Δ*mshE* strain was significantly decreased in all conditions compared to WTPtac0956 (Oneway ANOVA, Dunnett’s Multiple Comparison Test.)
(TIFF)

S1 Table Strains and plasmids used in this study. (DOCX)
