# Supplementary figures and images for: C-di-GMP Regulates Motile to Sessile Transition by Modulating MshA Pili Biogenesis and Near-Surface Motility Behavior in Vibrio cholerae
Source: PLoS Pathog. 2015 Oct 27;11(10):e1005068. doi: 10.1371/journal.ppat.1005068 (PMC4624765; doi:10.1371/journal.ppat.1005068)

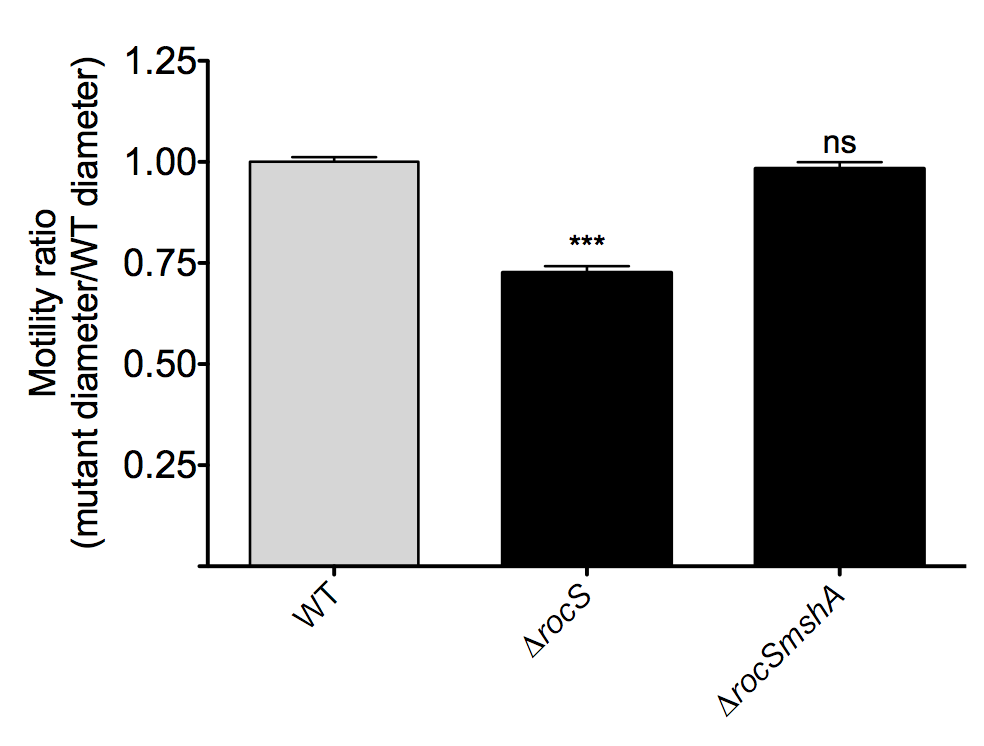

Supplement: S1 Fig — The diameters of migration zones of the WT and mutants were measured after 16 h of incubation at 30°C on LB soft agar motility plates and normalized to the motility of the WT strain. Three biological replicates were performed in quadruplicate. Statistical significance determined with Oneway ANOVA followed by Dunnett’s multiple comparison test comparing to the WT strain. (ns—not significant, *** p≤0.001) (TIFF) [file ppat.1005068.s001.tiff]

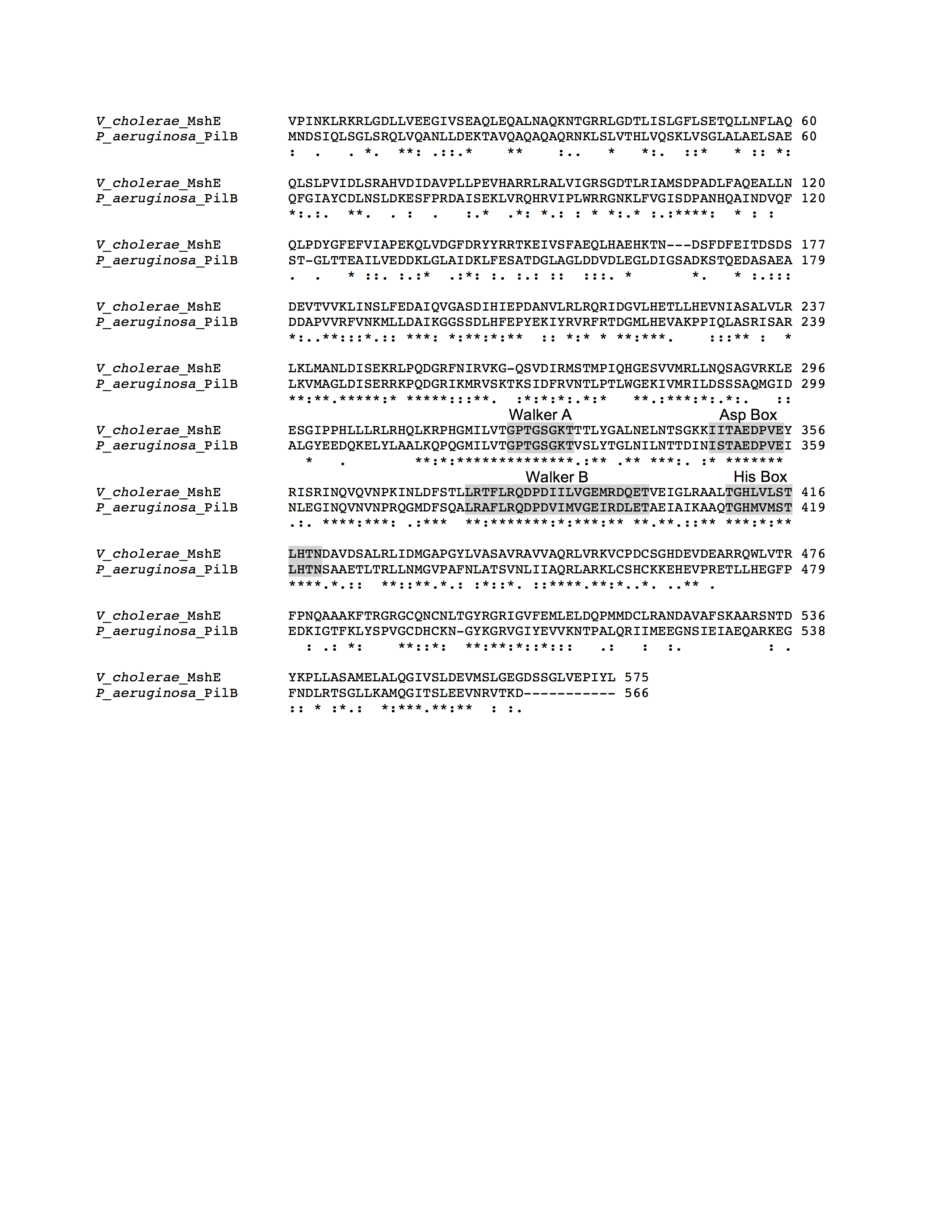

Supplement: S2 Fig — Amino acid alignment of MshE from V. cholerae and PilB from Pseudomonas aeruginosa indicates that there is 35% identity and 75% similarity. Stars indicate amino acid identity, colons indicate high similarity, and periods indicate similar amino acids. The conserved WalkerA, Asp Box, Walker B, and His Box domains are shaded. (TIFF) [file ppat.1005068.s002.tiff]

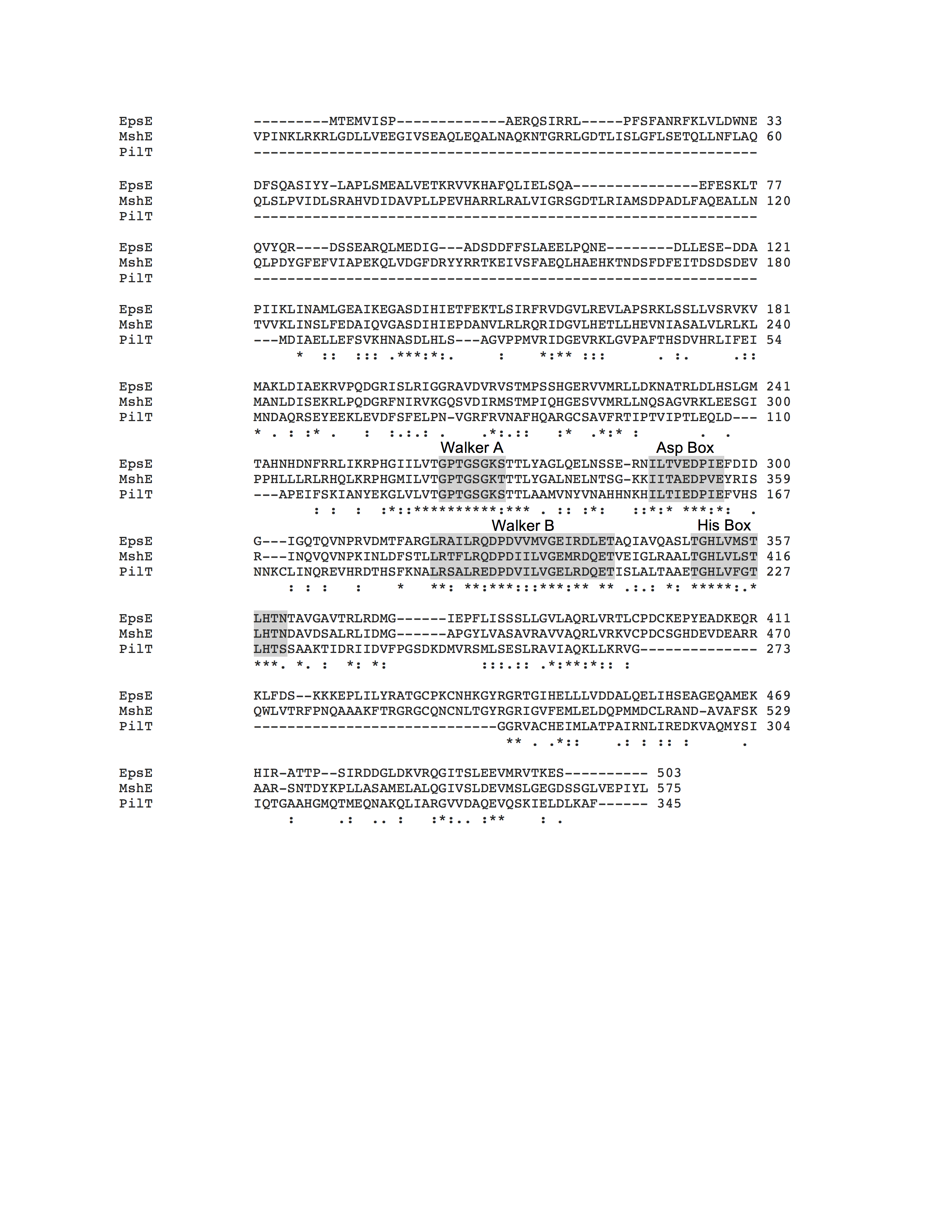

Supplement: S3 Fig — Amino acid alignment of MshE and EpsE indicates that there is 37% identity and 77% similarity, while PilT and EpsE share 26% identity and 73% similarity. Stars indicate amino acid identity, colons indicate high similarity, and periods indicate similar amino acids. The conserved WalkerA, Asp Box, Walker B, and His Box domains are shaded. (TIFF) [file ppat.1005068.s003.tiff]

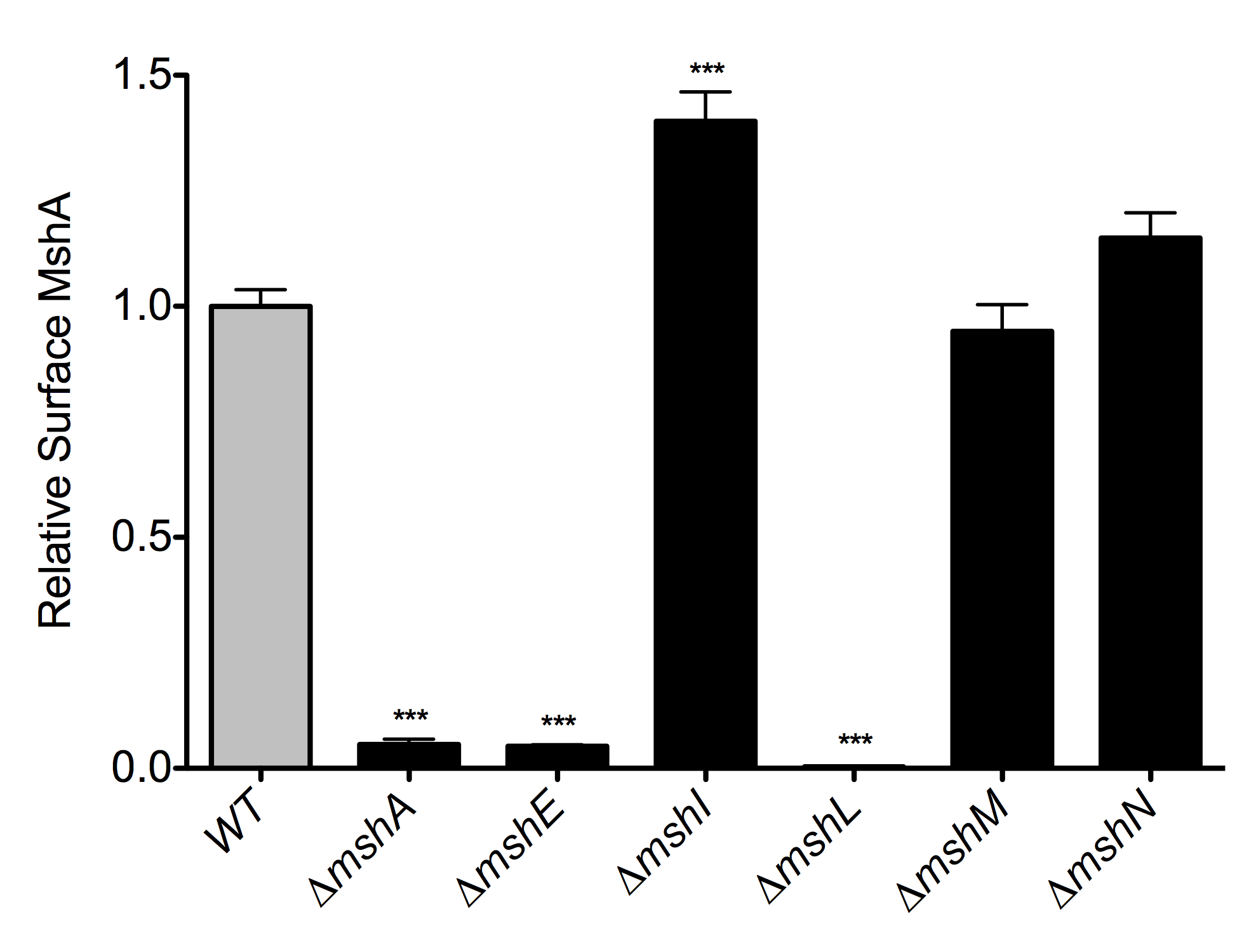

Supplement: S4 Fig — Strains with clean deletions of genes with multiple deletions in the secretory operon were generated and assayed for surface MshA pilus production. Deletion of mshA, mshE, and mshL abrogates production of surface MshA pili, while deletion of mshI, mshM, and mshN did not decrease MshA pilus production. Two biological replicates were assayed in quadruplicate and normalized to the average of the WT strain. Oneway ANOVA followed by Dunnett’s Multiple Comparison test compared to WT. (* p≤0.05, ** p≤0.01) (TIFF) [file ppat.1005068.s004.tiff]

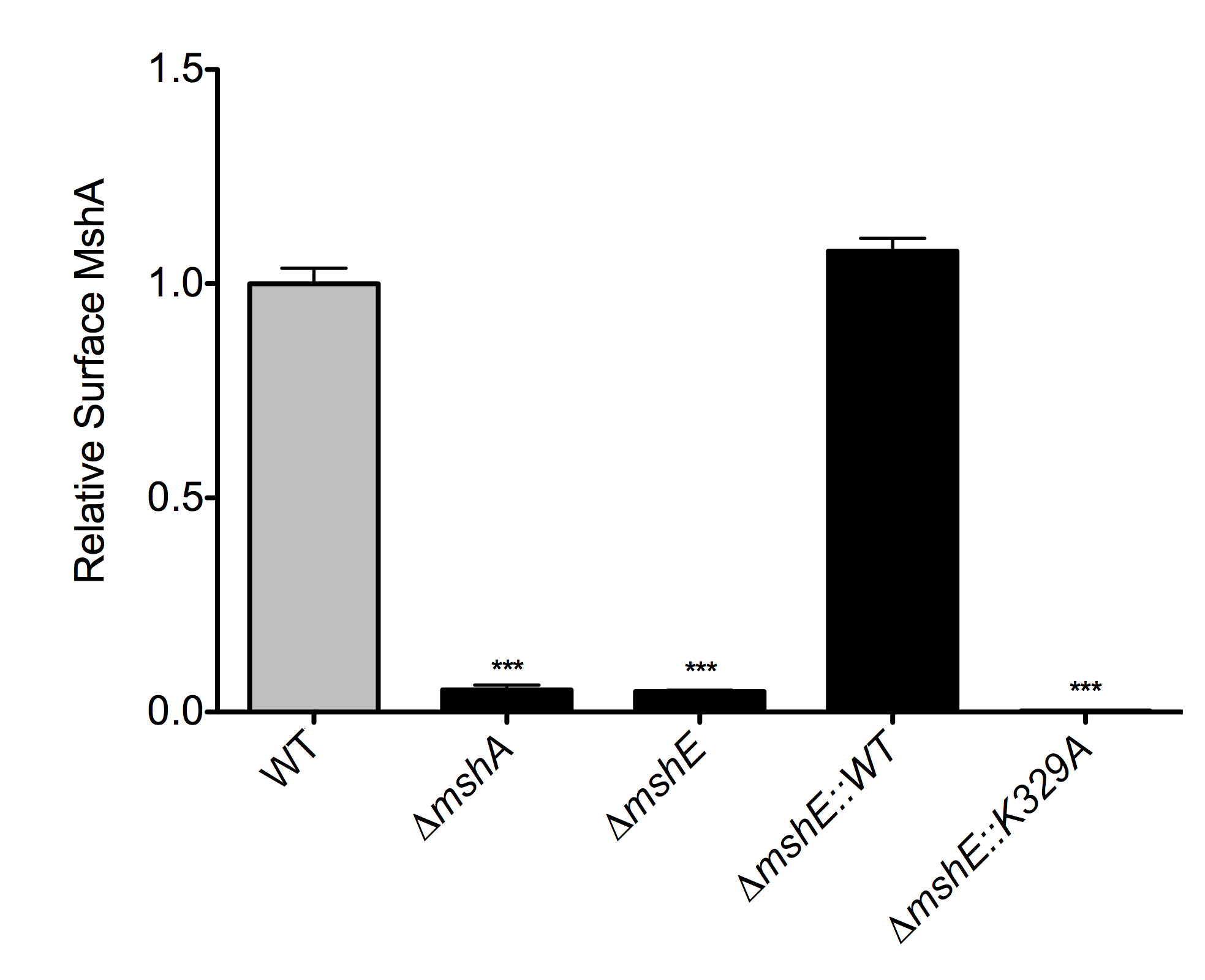

Supplement: S5 Fig — Surface pilin ELISAs indicate that replacing the chromosomal copy of mshE with a mutant of the lysine in the Walker A domain (K329A) abrogates pilus production. Three independent experiments were performed in triplicate. Significance was determined with an Oneway ANOVA followed by Dunnett’s Multiple Comparison Test compared to WT. (*** p≤0.001, all others not significant) (TIFF) [file ppat.1005068.s005.tiff]

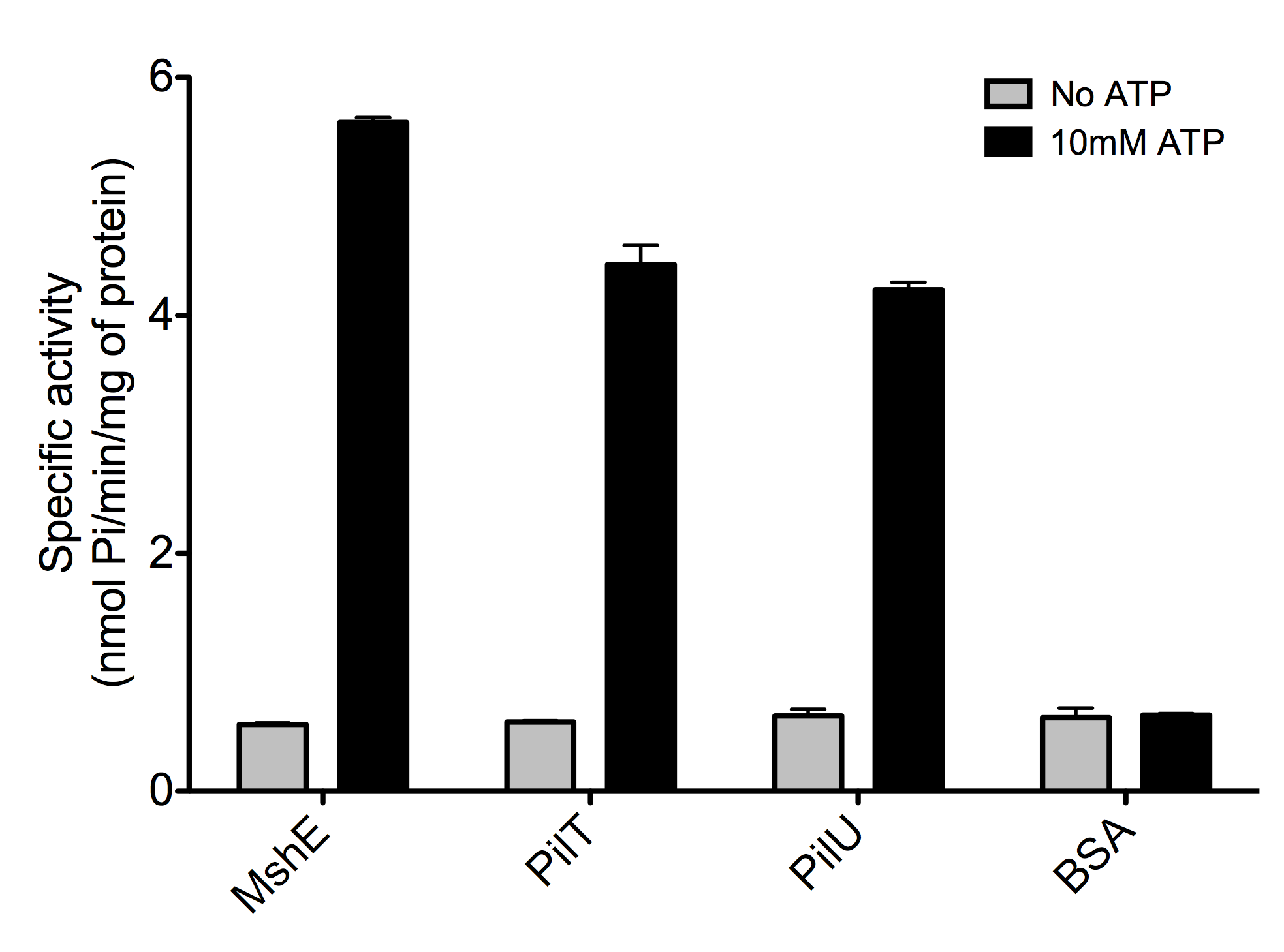

Supplement: S6 Fig — Production of inorganic phosphate from ATP by purified protein preparations was observed to determine functionality of purified proteins. The specific activity of a 5μM solution of protein with and without ATP is displayed after 30 minutes of incubation. Bovine Serum Albumin (BSA) was included as a negative control. Three independent experiments were performed in triplicate. (TIFF) [file ppat.1005068.s006.tiff]

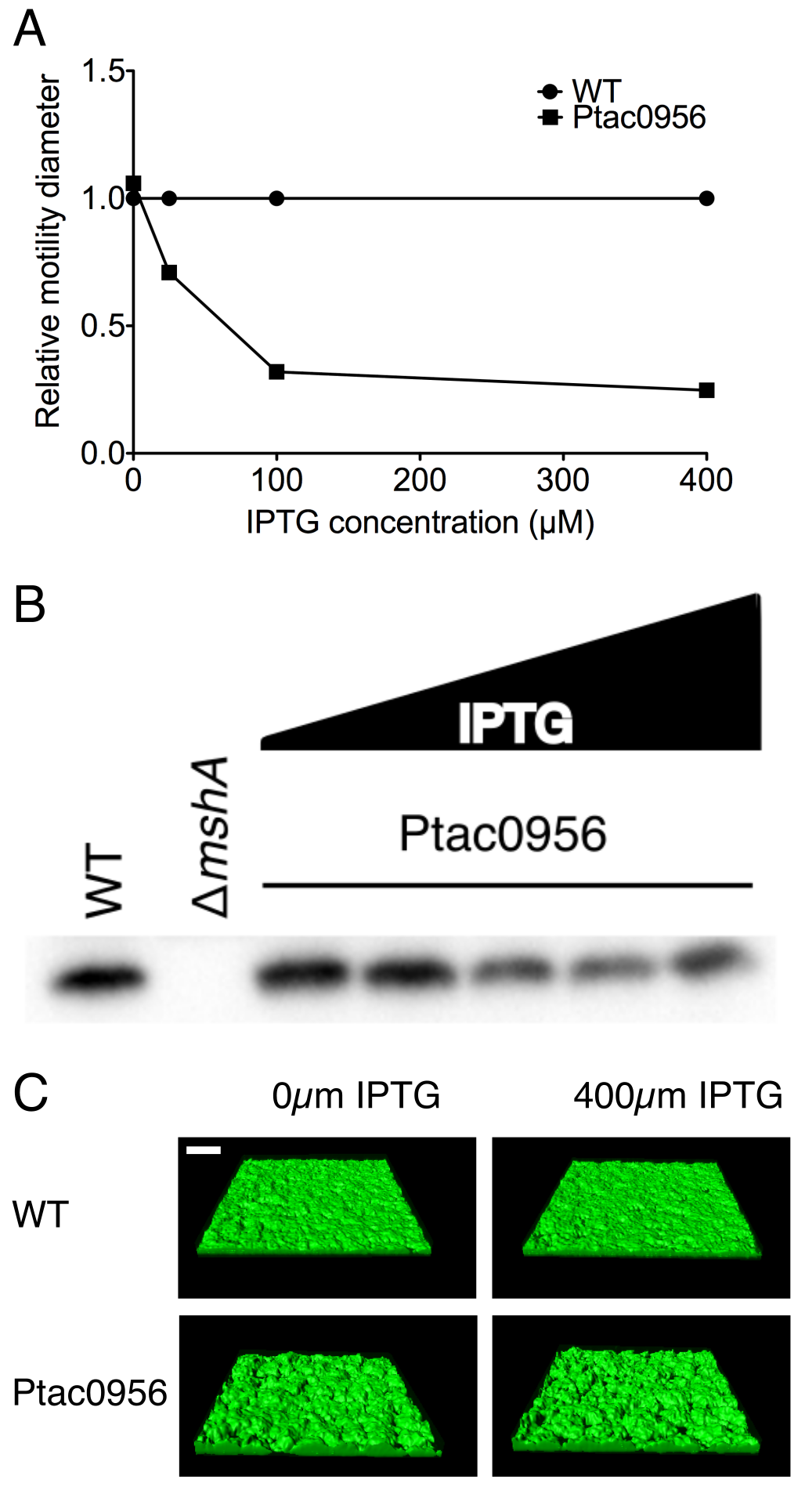

Supplement: S7 Fig — A. The diameter of migration zone of the WT and Ptac0956 strain were measured after 16 h of incubation at 30°C on LB soft agar motility plates containing a range of IPTG and normalized to the motility of the WT strain. Three biological replicates were performed in quadruplicate. B. Western blots detect MshA production over a range of IPTG concentrations (0, 6.25, 25, 100, 400μM) from cell pellets using α-MshA antibody. Blots were performed in triplicate, with one representative image included. C. Three-dimensional biofilm structures of the V. cholerae strains formed 24 h post inoculation in a flow cell system. Scale bar represents 40μm. Comstat analysis of two independent experiments in triplicate indicated that Ptac0956 with 400μM IPTG had significantly increased maximum thickness compared to WT (WT 13.42 ±1.50μm, Ptac0956 17.60±1.25μm p≤0.01). (TIFF) [file ppat.1005068.s007.tiff]

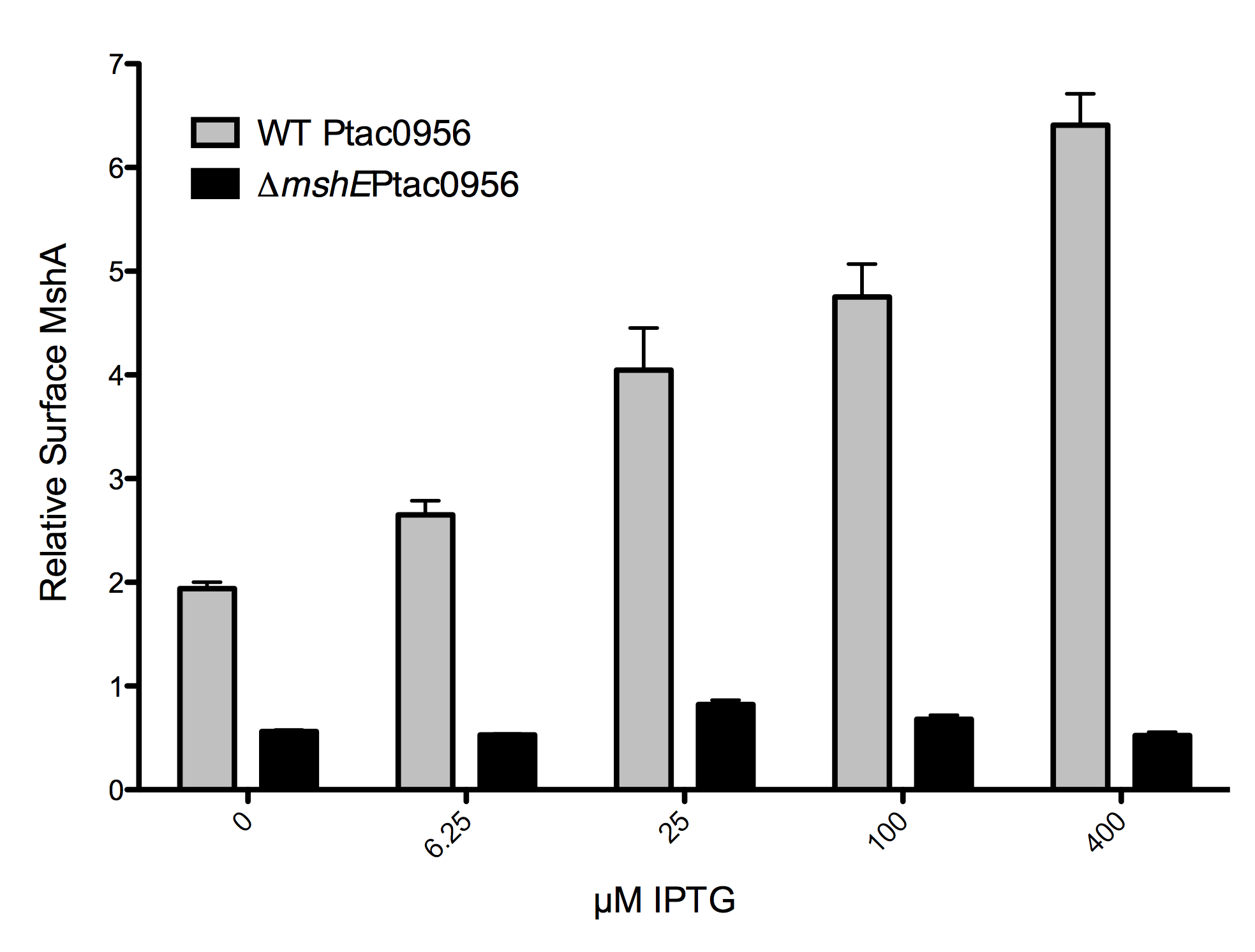

Supplement: S8 Fig — Expression of the DGC VCA0956 was induced with varying amounts of IPTG. Surface MshA pilin was determined by ELISA. Three biological replicates were tested in triplicate. Results were normalized to MshA production in the WT strain. Surface MshA pili production in the ΔmshE strain was significantly decreased in all conditions compared to WTPtac0956 (Oneway ANOVA, Dunnett’s Multiple Comparison Test.) (TIFF) [file ppat.1005068.s008.tiff]
