# Supplementary material for: C-di-GMP Regulates Motile to Sessile Transition by Modulating MshA Pili Biogenesis and Near-Surface Motility Behavior in Vibrio cholerae
Source: PLoS Pathog. 2015 Oct 27;11(10):e1005068. doi: 10.1371/journal.ppat.1005068 (PMC4624765; doi:10.1371/journal.ppat.1005068)
Supplement: S1 Table — (DOCX) [file ppat.1005068.s009.docx]

**S1 Table. Strains and plasmids used in this study.**

| Strain or plasmid | | Relevant genotype | Source |
| --- | --- | --- | --- |
| *E. coli* strains | | | |
|  | CC118λ*pir* | Δ(*ara*-*leu*) *araD* Δ*lacX74* *galE* *galK* *phoA20* *thi*-*1* *rpsE* *rpoB* *argE*(*Am*) *recA1* λ*pir* | [1] |
|  | S17-1λ*pir* | Tp^r^ Sm^r^ *recA thi pro* r_K_^-^ m_K_^+^ RP4::2-Tc::MuKm Tn*7* λ*pir* | [2] |
|  | SM10λ*pir* | thi thr leu tonA lacY supE recA (RP4-2-Tc::Mu) λpirR6K Km^r^ π^+^ | [3] |
| *V. cholerae* strains | | | |
|  | FY_VC_1 | *Vibrio cholerae* O1 El Tor A1552, WT, Rif^r^ | [4] |
|  | FY_VC_283 | Δ*mshA,* Rif^r^ | This study |
|  | FY_VC_337 | Δ*flaA,* Rif^r^ | [5] |
|  | FY_VC_354 | Δ*rocS*, Rif^r^ | [6] |
|  | FY_VC_745 | Δ*cdgJ*, Rif^r^ | [5] |
|  | FY_VC_8413 | Δ*cdgJ* Δ*mshA*, Rif^r^ | This study |
|  | FY_VC_8421 | Δ*rocS* Δ*mshA*, Rif^r^ | This study |
|  | FY_VC_8802 | Δ*pilT*, Rif^r^ | This study |
|  | FY_VC_8805 | Δ*pilU*, Rif^r^ | This study |
|  | FY_VC_8812 | Δ*mshE*, Rif^r^ | This study |
|  | FY_VC_9001 | Δ*cdgJ* Δ*pilT*, Rif^r^ | This study |
|  | FY_VC_9004 | Δ*cdgJ* Δ*pilU*, Rif^r^ | This study |
|  | FY_VC_9008 | Δ*cdgJ* Δ*mshE*, Rif^r^ | This study |
|  | FY_VC_9573 | WT mTn*7*-gfp, Rif^r^ Gm^r^ | [7] |
|  | FY_VC_9575 | Δ*mshA* mTn*7*-gfp, Rif^r^ Gm^r^ | This study |
|  | FY_VC_9581 | Δ*cdgJ* mTn*7*-gfp, Rif^r^ Gm^r^ | This study |
|  | FY_VC_9583 | Δ*cdgJ* Δ*mshA* mTn*7*-gfp, Rif^r^ Gm^r^ | This study |
|  | FY_VC_9584 | Δ*pilT* mTn*7*-gfp, Rif^r^ Gm^r^ | This study |
|  | FY_VC_9585 | Δ*pilU* mTn*7*-gfp, Rif^r^ Gm^r^ | This study |
|  | FY_VC_9586 | Δ*mshE* mTn*7*-gfp, Rif^r^ Gm^r^ | This study |
|  | FY_VC_9587 | Δ*cdgJ* Δ*pilT* mTn*7*-gfp, Rif^r^ Gm^r^ | This study |
|  | FY_VC_9588 | Δ*cdgJ* Δ*pilU* mTn*7*-gfp, Rif^r^ Gm^r^ | This study |
|  | FY_VC_9589 | Δ*cdgJ* Δ*mshE* mTn*7*-gfp, Rif^r^ Gm^r^ | This study |
|  | FY_VC_9917 | Insertion of the P_lacIq_-*lacI* and P_tac_ elements in front of VCA0956 in FY_VC_1, Rif^r^ | This study |
|  | FY_VC_9984 | Insertion of the P_lacIq_-*lacI* and P_tac_ elements in front of VCA0956 in FY_VC_8812, Rif^r^ | This study |
|  | FY_VC_10004 | Δ*mshE::mshE* WT, Rif^r^ | This study |
|  | FY_VC_10005 | Δ*mshE::mshE K329A*, Rif^r^ | This study |
|  |  |  |  |
| Plasmids | | | |
|  | pGP704*sacB*28 | pGP704 derivative, *mob*/*oriT* *sacB*, Ap^r^ | G. Schoolnik |
|  | pMAL-c5x | IPTG-inducible expression vector with N-terminal Maltose Binding Protein, Ap^r^ | NEB |
|  | pFY-12 | pGEX::*vpsT* | This study |
|  | pFY-291 | pGP704-sac28::Δ*mshA*, Ap^r^ | This study |
|  | pFY-1967 | pGP704-sac28::Δ*mshE*, Ap^r^ | This study |
|  | pFY-1975 | pGP704-sac28::Δ*pilT*, Ap^r^ | This study |
|  | pFY-1978 | pGP704-sac28::Δ*pilU*, Ap^r^ | This study |
|  | pFY-977 | pGP704-sac28::Δ*almEFG*, Ap^r^ | This study |
|  | pMMB67EH (ATCC® 37622™) | IPTG-inducible expression vector | ATCC |
|  | pHisGST Parallel1.1 | IPTG-inducible expression vector with N-terminal tags | Carrie Partch |
|  | pFY-3453 | pHisGST Parallel1.1::*mshE* | This study |
|  | pFY-3454 | pHisGST Parallel1.1::*pilT* | This study |
|  | pFY-3455 | pHisGST Parallel1.1::*pilU* | This study |
|  | pFY-3459 | pMMB67EH::*mshA* | This study |
|  | pFY-3495 | pGP704*sacB*28::P_tac_956-DR | This study |
|  | pFY-4163 | pHisGST Parallel1.1::*mshE* N terminus 1-180aa | This study |
|  | pUX-BF13 | oriR6K helper plasmid, *mob*/*oriT*, provides the Tn*7* transposition function *in trans*, Ap^r^ | [8] |
|  | pMCM11 | pGP704::mTn*7*-*gfp*, Gm^r^ Ap^r^ | M. Miller and G. Schoolnik |
|  | pTNSC189 | pSC189::MarinerTn, Km^r^Ap^r^ | [9] |

**References for S1 Table**

1. Herrero M, de Lorenzo V, Timmis KN. Transposon vectors containing non-antibiotic resistance selection markers for cloning and stable chromosomal insertion of foreign genes in gram-negative bacteria. J Bacteriol. 1990 Nov;172(11):6557–67.

2. de Lorenzo V, Timmis KN. Analysis and construction of stable phenotypes in gram-negative bacteria with Tn5- and Tn10-derived minitransposons. Meth Enzymol. 1994;235:386–405.

3. Taylor RK, Manoil C, Mekalanos JJ. Broad-host-range vectors for delivery of TnphoA: use in genetic analysis of secreted virulence determinants of *Vibrio cholerae*. J Bacteriol. 1989 Apr;171(4):1870–8.

4. Yildiz FH, Schoolnik GK. *Vibrio cholerae* O1 El Tor: Identification of a gene cluster required for the rugose colony type, exopolysaccharide production, chlorine resistance, and biofilm formation. Proc Natl Aad Sci USA; 1999 Mar 30;96(7):4028–33.

5. Liu X, Beyhan S, Lim B, Linington RG, Yildiz FH. Identification and characterization of a phosphodiesterase that inversely regulates motility and biofilm formation in *Vibrio cholerae*. J Bacteriol. 2010 Sep;192(18):4541–52.

6. Lim B, Beyhan S, Meir J, Yildiz FH. Cyclic-diGMP signal transduction systems in *Vibrio cholerae*: modulation of rugosity and biofilm formation. Mol Microbiol. 2006 Apr;60(2):331–48.

7. Beyhan S, Tischler AD, Camilli A, Yildiz FH. Differences in gene expression between the classical and El Tor biotypes of *Vibrio cholerae* O1. Infect Immun. 2006 Jun;74(6):3633–42.

8. Bao Y, Lies DP, Fu H, Roberts GP. An improved Tn7-based system for the single-copy insertion of cloned genes into chromosomes of gram-negative bacteria. Gene. 1991 Dec 20;109(1):167–8.

9. Chiang SL, Rubin, EJ. Construction of a *mariner*-based transposon for epitope-taggind and genomic targeting. Gene. 2002 Aug 21;296(2):179–85.
